# Versatile enhancement of the killing potential of anti-cancer agents achieved by peptide mimetics of the PCNA interface towards specialized DNA polymerases

**DOI:** 10.1038/s41419-025-07812-9

**Published:** 2025-07-08

**Authors:** Yiovana Verónica Okraine, María Belén de la Vega, Sofía Venerus Arbilla, Ginette Moyano, Agostina P. Bertolin, Horacio M. Pallarés, Lisa Wiesmüller, Sabrina F. Mansilla, Vanesa Gottifredi

**Affiliations:** 1 Fundación Instituto Leloir—Instituto de Investigaciones Bioquímicas de Buenos Aires, Consejo Nacional de Investigaciones Científicas y Técnicas (CONICET), Buenos Aires, Argentina; 2 The Francis Crick Institute, London, UK; 3 Stowers Institute, Kansas City, MO USA; 4 Department of Obstetrics and Gynecology, Ulm University, Ulm, Germany

**Keywords:** Peptides, Cancer

## Abstract

Cancer cells that survive chemotherapy achieve full DNA duplication despite the accumulation of damaged DNA triggered by chemotherapy. This happens because the synthesis of DNA at damaged sites is granted by tolerance events including translesion DNA synthesis (TLS), a process that promotes the use of specialized DNA polymerases (S-Pols) for DNA synthesis. Such a crucial role of S-Pols in the promotion of damaged DNA replication prompted analyses of the cell killing effects of individual S-Pols inhibitors. Because S-Pols can compensate for each other, a global inhibition of S-Pols needs to be designed and tested. Given that S-Pols are recruited to the replisome through their PCNA binding motif, we reasoned that global displacement of S-Pols will occur when delivering a peptide with a strong PCNA binding motif. The cyclin kinase inhibitor p21 contains the strongest PCNA binding motif. Therefore, we designed a peptide representing this C-terminal, PCNA interacting region (PIR) of p21. As hypothesized by us, the PIR peptide achieved global S-Pol displacement from PCNA-associated replication factories and enhanced the cancer cell killing potential of DNA damaging agents including cisplatin, hydroxyurea, olaparib and UV irradiation. Demonstrating strong versatility, the peptide also enhanced the cytotoxicity caused by agents that do not directly provoke DNA damage such as Chk1, ATR and Wee1 inhibitors. In all cases, disrupting the PCNA binding site within the PIR peptide was sufficient to dismantle its cell killing potential. Strengthening the concept, a less potent PIR, namely derived from S-Pol eta, efficiently displaced S-Pols from replication factories exacerbating cell killing by all agents tested. These results collectively indicate that simultaneous displacement of S-Pols from PCNA can be enforced by excess levels of PIR peptides. This strategy is demonstrably valid to enhance the cancer cell killing by different DNA-damaging agents.

## Introduction

Cancer cells cope with high levels of replication stress that arises from the accumulation of damaged DNA. Such chemical modifications of DNA provoke the stalling of replicative DNA polymerases (R-Pols). Auxiliary DNA replication processes, collectively known as DNA damage tolerance (DDT), have evolved to promote the utilization of damaged DNA regions as replication templates. Translesion DNA synthesis (TLS) is a DDT process that facilitates DNA replication continuity by prompting DNA synthesis by specialized DNA polymerases (S-Pols) capable of accommodating damaged DNA into their active sites [1]. S-Pols are recruited to the replisome through PCNA interacting regions (PIRs) in Pol eta (η), Pol kappa (κ) and Pol iota (ι) as well as an S-Pol interacting region and a BRCT domain in Rev1 [2, 3]. In addition, S-Pols have ubiquitin binding motifs, either UBM or UBZ, which enable their interaction with the DNA damage-induced ubiquitin moiety of PCNA, a post-translational modification triggered by fork stalling [4]. The switch from R-Pols to S-Pols is indeed prompted by the mono-ubiquitination of PCNA. Such post-translational modification favors mostly TLS at gaps left behind the replication fork, while Rev1 recruitment to replication forks promotes the switch from R- to S-Pols at stalled forks [1, 2, 5]. The activation of TLS at replication forks is accompanied by a change in the nuclear organization of S-Pols which re-group into discrete nuclear foci that localize at sites of DNA synthesis [6–8]. The need of S-Pols for the maintenance of efficient DNA synthesis in conditions of augmented DNA damage is evidenced by the shortening of nascent DNA tracks after downregulation of each single S-Pol [1]. Hence, TLS by S-Pols is required to prevent excess fork stalling in cells challenged with DNA damaging agents.

S-Pols are specialized in terms of the type of DNA-damage they accommodate in their active site. For example, Pol η is the preferred choice for cyclobutane pyrimidine dimers (CPDs) caused by ultraviolet irradiation (UV) but is not equally advantageous as a choice for other types of damaged DNA templates [9]. Similarly, Pol κ is needed for TLS at benzo[a]pyrene diol epoxide (BPDE)-DNA adducts [10, 11]. After elimination of the S-Pol of choice, TLS efficiency is impaired but is not fully eliminated. For example, suboptimal DNA replication that results from the loss of S-Pols such as Pol η in UV-irradiated samples is followed by inefficient TLS by Pol ι and repriming events by PrimPol [12, 13].The relevance of S-Pols for the survival of cells challenged by DNA damage is also revealed when analyzing the effect of chemotherapy in patients. For example, in cells that acquire resistance to cisplatin (CDDP), the levels of Pol η are frequently increased [14]; hence suggesting that excess TLS by Pol η protects cancer cells from CDDP-mediated cell killing. Because of such a contribution of Pol η to the adaptation of DNA replication to cancer therapy, it has been suggested that TLS inhibition is a potential strategy worth exploring [15].

In the last decade, several TLS inhibitors were designed and tested [16, 17]. Specifically, inhibitors of Pol η, Pol κ and Rev1 were reported to synergize with chemotherapeutic agents such as CDDP or mitomycin C (MMC). Those inhibitors have not yet reached clinical trials but some encouraging pre-clinical data has been reported for the Rev1 inhibitor JH-RE-06 [18, 19]. However, the versatility of TLS inhibition in terms of its capacity to augment the cancer cell killing ability of a broad range of anti-cancer agents has not been tested yet.

While TLS inhibitors may displace a specific S-Pol from the replisome, there is no strategy that simultaneously blocks different types of S-Pols. The C-terminus of the cyclin kinase inhibitor, p21, best known for its ability to inhibit cyclin kinases through its N-terminal domain, binds PCNA at the same docking site also used by S-Pols [20]. In fact, overexpression of a p21 mutant that resists UV-mediated degradation is sufficient to prevent the recruitment of all S-Pols to replication factories, augmenting replication stress and enforcing cell killing by UV irradiation [21, 22]. Such an ability of p21 depends on its interaction with PCNA but it is unclear if the isolated PCNA interacting motif of p21 is sufficient to achieve S-Pol displacement and enhanced cancer cell killing. Here we show that a short C-terminal peptide of 26 amino acids encoding the PCNA interacting region of p21 (sPIR^p21^) is equally effective as the full-length p21 at inhibiting the nuclear focal organization of S-Pols on chromatin. Similarly, the full-length p21 and the sPIR^p21^, but not sPIR^p21ΔP^, a mutated version that does not bind to PCNA, cause nascent DNA shortening after UV irradiation. Remarkably, sPIR^p21^ but not sPIR^p21ΔP^ potentiates the cell killing effect of various DNA damaging treatments including UV, hydroxyurea (HU), CDDP and olaparib (Ola). The sPIR^p21^ also augments cancer cell killing by agents that do not directly induce DNA damage such as Chk1, ATR and Wee1 inhibitors. Hence, the sPIR^p21^ peptide is a very versatile enhancer of cell killing by different chemotherapeutic agents. Such effects were equally observed with another PIR derived from Pol η, implying that PIRs in general and not just the specific sequence of PIR^p21^ potentiate the cell killing effect of chemotherapy. These results suggest that the disruption of the S-Pol-PCNA interaction may be key to potentiate several cancer therapeutic schemes.

## Results

### The PCNA binding motif of p21 interferes with the upregulation of TLS-associated events to the same extent as full-length p21

The cyclin kinase inhibitor, p21, binds to the PCNA region that interacts with S-Pols. In fact, we have previously demonstrated that the strong PCNA-interacting region (PIR) of p21 inhibits TLS and increases cell death after UV [22]. This effect was achieved by a sp21 version of p21 that expressed a 6-myc-tag on its N-terminus that prevents p21 proteolysis [23, 24]. Importantly, sp21 and other p21 constructs used in this study differ from endogenous p21 in that they harbor a disrupted CDK-binding motif. Such a mutation allows cycle progression despite the augmentation of the levels of p21 mutants [21]. In that way, S phase progression in both control and p21 expressing samples allows the encounter of replication forks with damaged DNA and, consequentially, the need of activation of TLS events at those damage-based replication barriers. Notwithstanding the 6-myc stabilizing sequence, sp21 still contains a degron motif that triggers its degradation when associated with PCNA [25]. To test whether the TLS inhibitory features of sp21 augment when such degron is disrupted, we introduced a point mutation in the degron sequence of p21 and sp21 (Supplementary Fig. 1A). All p21 mutants localized to the nucleus and were expressed at levels in the range of the ones reached after daunorubicin treatment (a chemotoxin that causes a p53-mediated accumulation of endogenous p21 (Supplementary Fig. 1B, C). As previously shown by us [21], such an overexpression of p21 did not impair the transit trough S phase. Moreover, the disruption of the degron in p21 did not modify the ability of cells to transit S phase (Supplementary Fig. 1D, E). To determine the relevance of such degron sequence for p21 proteolysis, the half-life of different p21 versions was determined. The disruption of the degron sequence in p21ΔDeg increased the half-life of p21 in untreated conditions (Supplementary Fig. 1F, G), but did not confer stability to p21 in UV-treated conditions (Supplementary Fig. H). However, when the stabilizing 6-myc-tag was added, the disruption of the degron in sp21ΔDeg did not further modify the protein half-life both before and after UV (Supplementary Fig. 1I–K). When evaluating Pol η focal organization, the quick post-UV degradation of p21 and p21ΔDeg was sufficient to allow an efficiency of focal organization similar to that of the control sample. Instead, sp21 and sp21ΔDeg caused a similar inhibition of Pol η focal organization (Supplementary Fig. 1L, M). Hence, we concluded that the mutation of the degron sequence within the PCNA interacting motif does not affect the TLS inhibiting functions of p21; therefore, we did not include the degron mutated p21 and sp21 versions in further experiments of this study.

**Fig. 1:**
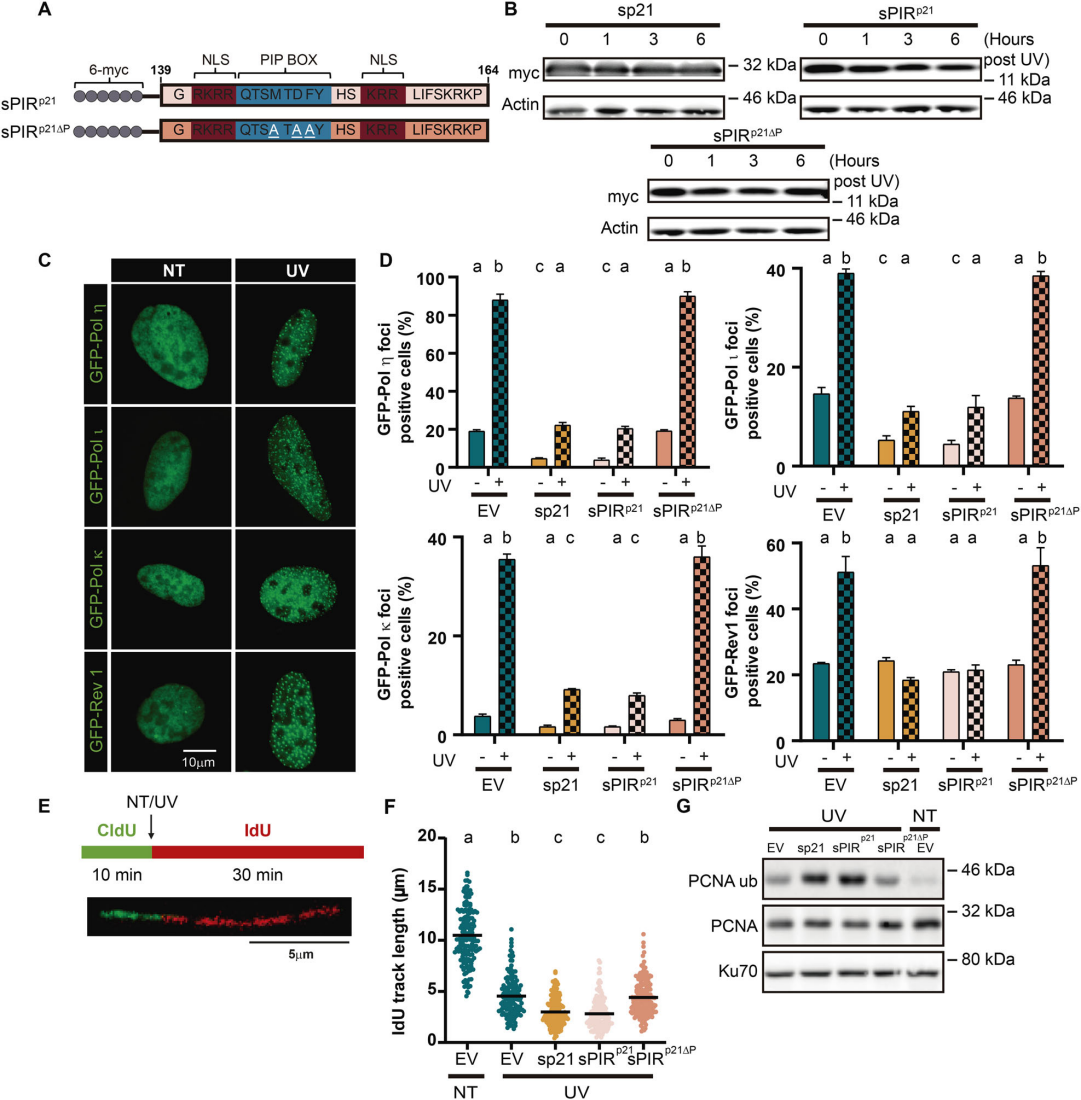
sPIR^p21^ is as efficient as full-length p21 in impairing TLS-associated events after UV treatment. **A** Schematic presentation of the sPIR^p21^ variants used in this study encoding amino acid residues 139 to 164 of p21, fused to a 6-myc-tag at the N-terminus. The eight amino acid sequence corresponding to the PIP box are within the blue box. The bipartite NLS is represented by the dark red box. To generate the sPIR^p21ΔP^, three point mutations in the sPIR^p21^ sequence were introduced (M147A, D149A, F150A) to disrupt the interaction of p21 with PCNA [23]. NLS: nuclear localization signal. For further details about the mutants used, see the “Materials and methods” section. **B** Western blot of lysates obtained from U2OS cells transduced with sp21, sPIR^p21^ or sPIR^p21ΔP^ lentiviral vector treated with UV (40 J/m^2^) 48 h after transduction. Whole cell extracts were prepared at the indicated time points. Anti-myc antibodies were used to detect myc-tagged p21. Actin was used as a loading control. A representative experiment is shown. *N* = 2 (*N* number of independent experiments). **C** Representative images of nuclei transfected with GFP-Pols under untreated (NT) and UV irradiated (UV) conditions. **D** Percentage (mean ± SD) of U2OS cells with detectable nuclear foci of the indicated DNA polymerase. Samples were transfected with the indicated GFP-Pols and 5 h later transduced with EV, sp21, sPIR^p21^ or sPIR^p21ΔP^. 48 h later, cells were treated with UV (40 J/m^2^) and fixed 4 h later. Transduced cells were detected by using an anti-myc specific antibody, and the focal organization of GFP-Pols was quantified in 200 nuclei positive for both GFP and myc. *N* = 3 (Statistics: one-way ANOVA, Tukey post test). The letters on top of the columns in this graph, as well as in all other graphs in this study with statistical analysis, mark grouped samples that are not significantly different. Samples not sharing a letter are significantly different. For more information about statistical analysis, see “Materials and methods”. **E** Labeling protocol used for the DNA fiber spreading assay. Bottom: representative image of a single bicolor DNA fiber of U2OS cells. **F** Quantification of IdU track length from U2OS cells transduced with the indicated vectors. Median is shown in black. Cells were either mock (NT) treated or treated with UV (40 J/m^2^). ~200 fibers/sample were analyzed. Data shown for the condition NT EV is shown again in Fig. 2I. *N* = 2 (Statistics: Kruskal–Wallis, Dunn post test). **G** Western blot of U2OS cells transduced with the indicated vectors. 48 h after transduction with the indicated vectors, cells were either mock treated (NT) or treated with UV (40 J/m^2^). 4 h later samples were harvested for whole cell extraction. PCNA ubiquitination and PCNA were detected using specific antibodies against ubiquitinated PCNA and PCNA respectively. Ku70 was used as a loading control. A representative experiment is shown. *N* = 2.

We then attempted to determine if the small PIR region of p21 is sufficient to achieve the same TLS inhibition reached by the full-length sp21. We generated lentiviral vectors encoding sp21 (Supplementary Fig. 1A), a 6-myc-tagged peptide comprising the 26 C-terminal amino acid residues of p21 (sPIR^p21^) (Fig. 1A) that encompasses the PCNA binding region and flanking sequences which are relevant for the strength of its interaction with PCNA [26, 27]. As a control, we also generated an sPIR^p21ΔP^ mutant with 3 point mutations known to disrupt the PCNA-p21 interaction [21, 23] (Fig. 1A). Since all mutants maintain the bipartite nuclear localization signal localized in its C-terminus, sp21, sPIR^p21^ and sPIR^p21ΔP^ were found localized to the nucleus (Supplementary Fig. 2A). The transduction of p21 expressing vectors was efficient and p21 levels were similar (Supplementary Fig. 2B, C). All mutants remained stable after UV irradiation (Fig. 1B). Next, we set up a co-transduction with lentiviral particles encoding the above-mentioned vectors and GFP-Pol η, GFP-Pol ι, GFP-Pol κ and GFP-Rev1 (Supplementary Fig. 2D–H), which have been extensively characterized in terms of their ability to be recruited to replication factories after exposure of cells to different DNA damaging treatments [22, 28]. The peptide sPIR^p21^ but not sPIR^p21ΔP^ prevented nuclear foci formation of the S-Pols to an extent that corresponded to the effect of sp21 (Fig. 1C, D). In line with the effects of sPIR^p21^ on S-Pols nuclear organization, sPIR^p21^ interacted with PCNA according to proximity ligation assay (PLA) and such an interaction was severely impaired in samples were sPIR^p21ΔP^ was expressed (Supplementary Fig. 2I, J). To focus on a parameter revealing a consequence of suboptimal TLS events, we analyzed the effect of the sPIR^p21^ on the efficiency of nascent DNA synthesis. We quantified the length of nascent DNA tracks in samples treated with UV. While tracks shorten when comparing untreated and UV-treated samples, their remaining length is known to depend on the presence of S-Pols aiding DNA replication post-UV [1]. Therefore, even further shortening reveals impaired TLS events. Indeed, the length of nascent post-UV DNA tracks was shorter in sp21 expressing samples than in those corresponding to the EV control condition. A similar track shortening was revealed when expressing sPIR^p21^ but not sPIR^p21ΔP^ (Fig. 1E, F) suggesting that the sPIR^p21^ was as efficient as sp21 in impairing TLS events, implying that the sp21 effect was fully dependent on PCNA binding. We also observed an increase in PCNA ubiquitination levels in UV-treated sp21 or sPIR^p21^-expressing but not sPIR^p21ΔP^-expressing samples (Fig. 1G), which indicates that suboptimal TLS may be coupled with persistent stalling of replication forks at DNA damage or decisions towards new DDT pathway choices [1, 29]. In conclusion, the TLS inhibitory capacity of p21 is fully dependent on a small 26-residue PCNA-binding region located in its C-terminus.

### sPIR^p21^, but not sp21, reduces cell proliferation in undamaged conditions

**Fig. 2:**
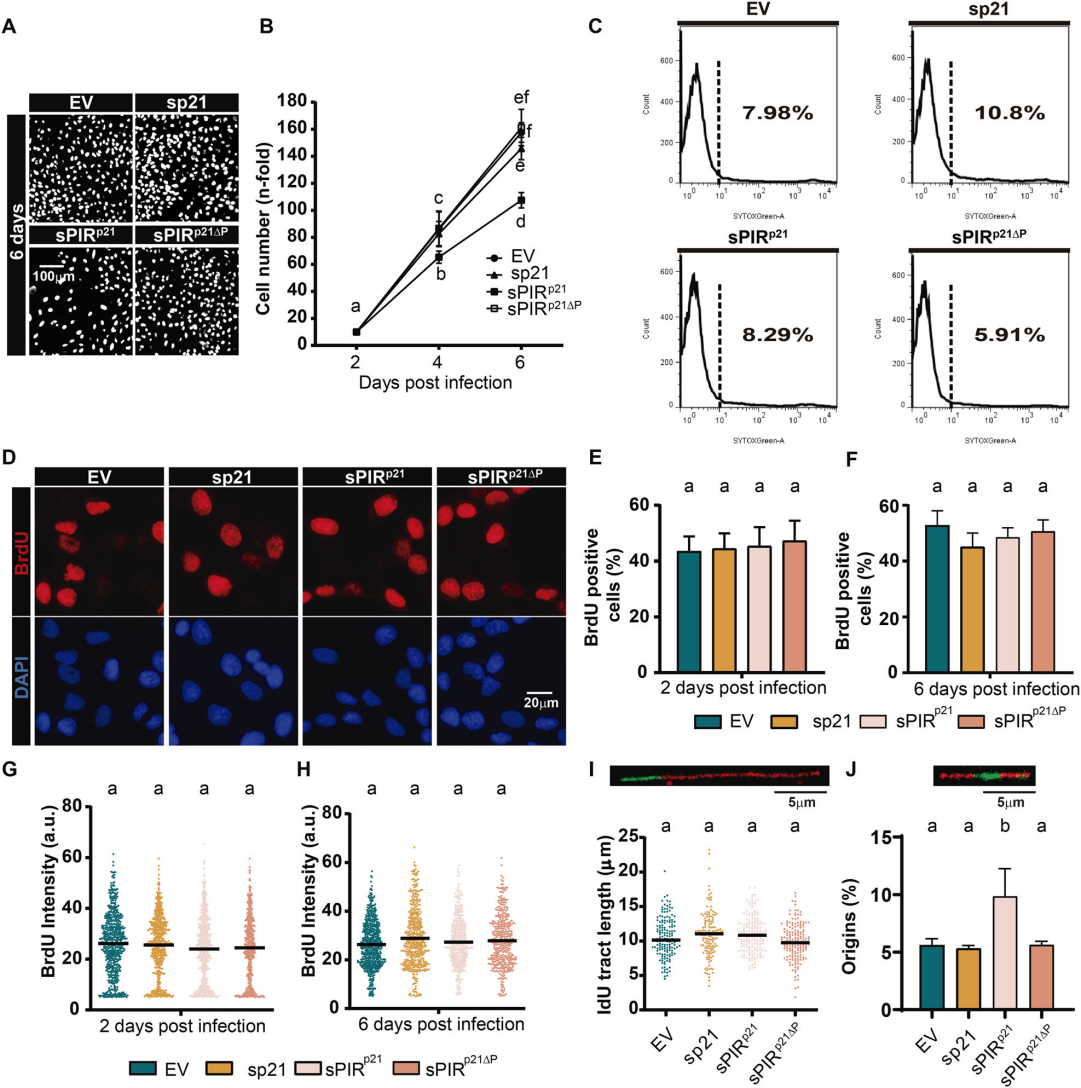
In the absence of DNA damage, sPIR^p21^ but not sp21 reduces cell count in a manner that is dependent on PCNA. **A** Representative images of U2OS cells transduced with the indicated vectors. Nuclei were visualized after DAPI staining. **B** Cell number (mean ± SD) of U2OS cells transduced as indicated and fixed at the indicated time points. The number of cells is expressed relative to the cell number of a sample transfected with the same vector, fixed 2 days post transduction. The total cell number in 3 wells from a 96-well plate was counted for each condition. *N* = 3 (Statistics: one-way ANOVA, Tukey post test). **C** FACS analysis of SYTOX green-stained U2OS cells 6 days after transduction as indicated in the panel. The dotted line represents the separation between the cells that stain positive or negative for SYTOX Green. On the right side of the dotted line, the numbers indicate the percentages of the cell population displaying high intensity of SYTOX green. A representative experiment is shown. *N* = 3. **D** Representative images of U2OS cells transduced as indicated in the panel. 48 h after transduction, cells were pulse-labeled with BrdU for 15 min before fixation, denaturation and incubation with an anti-BrdU specific antibody. **E** Quantification (mean ± SD) of BrdU positive cells 2 days after transduction. Cells were pulse-labeled as indicated in (**D**). At least 300 nuclei/sample were analyzed. *N* = 3 (Statistics: one-way ANOVA, Tukey post test). **F** Quantification (mean ± SD) of BrdU positive cells 6 days after transduction. Cells were pulse-labeled as indicated in (**D**) and quantified as in (**E**). **G** Quantification of BrdU intensity of the experiments shown in (**E**). Median is shown in black. At least 500 positive BrdU cells were analyzed. *N* = 3 (Statistics: Kruskal–Wallis, Dunn post test). **H** Quantification of BrdU intensity of experiments shown in (**F**). Median is shown in black. At least 400 positive BrdU cells were analyzed. *N* = 3 (Statistics: Kruskal–Wallis, Dunn post-test). **I** Quantification of IdU track length. A representative image of a DNA fiber is shown on the top portion of the panel. After incorporation of CldU for 10 min and IdU for 30 min, samples were processed according to the DNA fiber spreading protocol and the IdU track length was measured. Median is shown in black. ~200 fibers/sample were analyzed. Data corresponding to the NT EV condition is shown also in Fig. 1F. *N* = 2 (Statistics: Kruskal–Wallis, Dunn post-test). **J** Quantification of origin firing frequency. A representative image of a DNA fiber containing an origin is shown on the top portion of the panel. The percentage of origin firing (mean ± SD) was determined in the same samples used in **I** as the relative number of origins [(red-green-red + red only fibers)/total fibers]. ~400 fibers/samples were analyzed. Images used to quantify origins are the same as those used in Fig. 2I. *N* = 2 (Statistics: one-way ANOVA, Tukey post test).

We and others have shown that the PCNA binding region of p21 does not impair proliferation under unperturbed conditions [21, 22, 30], which is consistent with the absence of an effect by sp21 here (Fig. 2A, B). Intriguingly, sPIR^p21^, but not sPIR^p21ΔP^, caused a reduction in the cell number, which became increasingly evident when cells expressing sPIR^p21^ underwent multiple rounds of duplication (Fig. 2A, B). We first tested whether the reduction in cell number was associated with cell death and performed SYTOX green exclusion experiments that indicated that it was not the case (Fig. 2C). To determine if the proliferation decrease caused by sPIR^p21^ was associated with a defect in S phase progression, we quantified both the percentage and intensity of BrdU incorporation at early and late time points which did not reveal such a defect (Fig. 2D–H). Consistent with the previous result, nascent DNA tracks were also not modulated by sPIR^p21^ (Fig. 2I). Collectively, these data indicate that under unperturbed conditions, the ability of sPIR^p21^ to reduce the cell number is unlikely to be associated with TLS inhibition, which is very different from the scenario after DNA damage. Intriguingly however, we reveal an S phase specific parameter that is selectively modulated by sPIR^p21^ but not sp21, namely the frequency of origin firing (Fig. 2J). Such a result suggests that, in a manner unrelated to track-length associated events, sPIR^p21^, but not sp21, induces subtle but detectable alterations in the DNA replication program that cause a reduction in cell number in untreated cells.

**Fig. 3:**
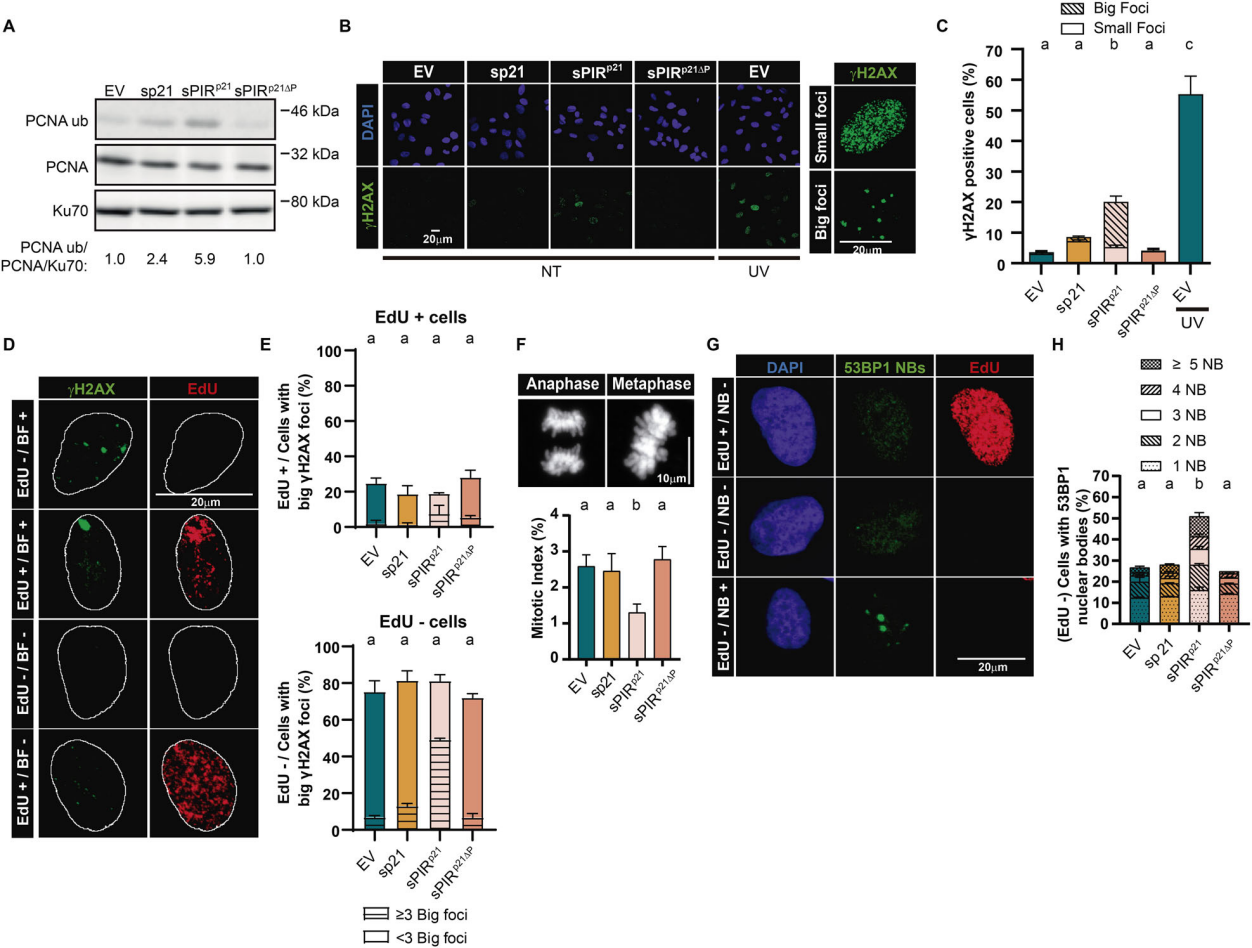
sPIR^p21^ increases replication stress and negatively impacts on the M phase transition in the absence of DNA damage. **A** On top: Western blot of U2OS cells 48 h after transduction with EV, sp21, sPIR^p21^ or sPIR^p21∆P^. PCNA ubiquitination and PCNA were detected using specific antibodies against ubiquitinated PCNA and PCNA respectively. Ku70 was used as a loading control. A representative image is shown. Below: densitometry quantification of the bands shown above. *N* = 2. **B** On the left: Representative images showing levels of γH2AX staining in U2OS cells 48 h after the transductions indicated in the panel. UV irradiation (5 J/m^2^) of the EV sample was used as a positive control of the levels of induction of γH2AX by damaged DNA. On the right: representative images of single nuclei showing the distribution of γH2AX in small and big foci. **C** Quantification (mean ± SD) of U2OS nuclei positive for small (empty boxes) and big γH2AX foci (striped boxes). 200 cells/sample were analyzed. *N* = 3 (Statistics: one-way ANOVA, Tukey post test). **D** Representative images of U2OS nuclei positive (+) or negative (−) for EdU and positive (+) or negative (−) for big γH2AX foci (BF). White line delimits the nuclei area. DAPI channel data was used to create a mask in order to generate the white line. **E** Percentages (mean ± SD) of U2OS nuclei positive or negative for EdU for the population of cells with big γH2AX foci 48 h after transduction as indicated in the panels. At least 50 nuclei with big γH2AX foci/sample were analyzed. *N* = 3 (Statistics: one-way ANOVA, Tukey post test). **F** On top: Representative images of mitotic U2OS cells. Maximum intensity projection of Z-stack is shown. Bottom: percentages (mean ± SD) of mitotic cells out of U2OS cells transduced as indicated in the panel. At least 1000 cells/sample were analyzed. *N* = 4 (Statistics: one-way ANOVA, Tukey post test). **G** Representative images of U2OS nuclei positive (EdU +) or negative (EdU −) for EdU staining and positive (NB +) or negative (NB −) for 53BP1 nuclear bodies. **H** Percentages (mean ± SD) of EdU negative (EdU −) nuclei with 1 to 5 or more 53BP1 nuclear bodies (boxes on top) from U2OS cells 48 h after transduction as indicated in the panels. At least 150 EdU negative nuclei/sample were analyzed. *N* = 2 (Statistics: one-way ANOVA, Tukey post test).

Consistent with the reduction in cell number, we obtained evidence for an altered DNA damage response (DDR) in unperturbed cells expressing sPIR^p21^ but not sp21 or sPIR^p21ΔP^. First, the levels of PCNA mono-ubiquitination increased after sPIR^p21^ expression (Fig. 3A). Second, we also noticed the accumulation of cells with a peculiar focal organization of phosphorylated histone H2AX (γH2AX), creating a pattern of big foci that selectively accumulated in sPIR^p21^-expressing cells (Fig. 3B, C) which were outside S phase (Fig. 3D, E). Third, we observed a reduction in the mitotic index and an increase in 53BP1 nuclear bodies in G1 only in samples expressing sPIR^p21^ (Fig. 3F–H). All these alterations very likely precede the reduction in the number of cells expressing sPIR^p21^ (Fig. 2A, B). In conclusion, these data suggest that the peptide sPIR^p21^ delays cell cycle progression by modulating parameters outside of S phase which are not affected by the full-length sp21 protein.

### The peptide sPIR^p21^ derived from p21 is a versatile enhancer of DNA damaging treatments that cause activation of TLS

**Fig. 4:**
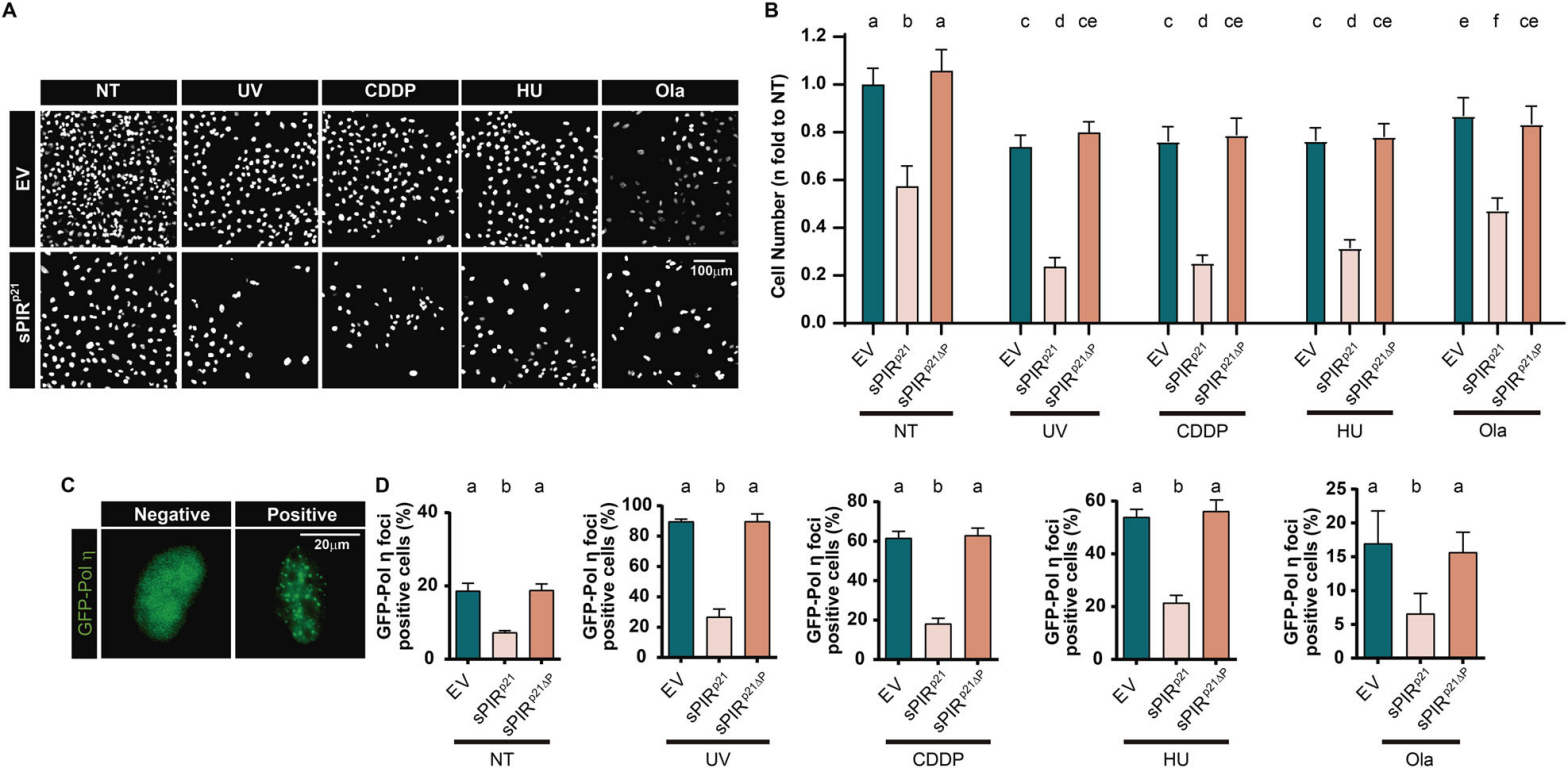
sPIR^p21^ cooperates with DNA damaging agents in killing cancer cells. **A** Representative images of DAPI-stained U2OS cells transduced with either EV or sPIR^p21^ particles. 48 h later, cells were either mock treated (NT) or treated with UV (5 J/m^2^), CDDP (1 μM), HU (0.25 mM) or olaparib (0.65 µM) and fixed 6 days later. **B** Cell number (mean ± SD) expressed as fold changes with respect to the EV transduced, mock treated (NT) U2OS cells shown in (**A**). The total cell number in 3 wells from a 96-well plate was quantified for each condition. *N* = 3 (Statistics: one-way ANOVA, Tukey post test). **C** Representative images of U2OS cells transfected with GFP-Pol η, showing either its pan-nuclear (negative) or its focal (positive) organization. **D** Percentage (mean ± SD) of U2OS cells with nuclear GFP-Pol η foci. U2OS cells were transfected with GFP-Pol η and 5 h later transduced with EV, sPIR^p21^ and sPIR^p21ΔP^. 48 h later, cells were mock-treated (NT) or treated with UV (40 J/m^2^), CDDP (33 μM), HU (2 mM) and fixed 5 h later. Cells treated with Ola (0.65 µM) were fixed 24 h after the addition of Ola. At least 200 nuclei positive for both GFP and myc/sample were analyzed. *N* = 3 (Statistics: one-way ANOVA, Tukey post test).

Having established that sPIR^p21^ affects cell proliferation under unperturbed conditions, we wondered if sPIR^p21^ enhances the cell killing by chemotherapeutic agents that damage DNA templates in a manner that augments the dependency of DNA replication on TLS activation. We quantified the number of cells in the presence or absence of sPIR^p21^, using the following agents: UV irradiation which induces CPDs and other DNA lesions [31, 32]; CDDP which triggers the formation of DNA adducts [33]; HU, which blocks dNTP synthesis, impairing DNA replication [34]; Ola, which causes the accumulation of protein-DNA adducts which may favor TLS events. While each single agent negatively affected the cell number, the combination of such chemotoxins and sPIR^p21^ reduced it even further. This effect fully depended on the PCNA binding ability of sPIR^p21^ as revealed by a failure of the sPIR^p21ΔP^ to reduce the cell number (Fig. 4A, B). Increased cell killing was also evidenced when normalizing data to the untreated condition, hence demonstrating synergy between sPIR^p21^ and DNA damaging treatments (Supplementary Fig. 3). Strengthening the notion that cell killing was connected to TLS inhibition by sPIR^p21^, we found that the focal organization of Pol η was impaired by sPIR^p21^ but not sPIR^p21ΔP^ (Fig. 4C, D). In conclusion, sPIR^p21^ enhanced the anti-proliferative effect of chemotoxins with the ability to cause the accumulation of replication barriers.

### sPIR^p21^ potentiates the cell killing by agents that trigger DDR unrelated to TLS

**Fig. 5:**
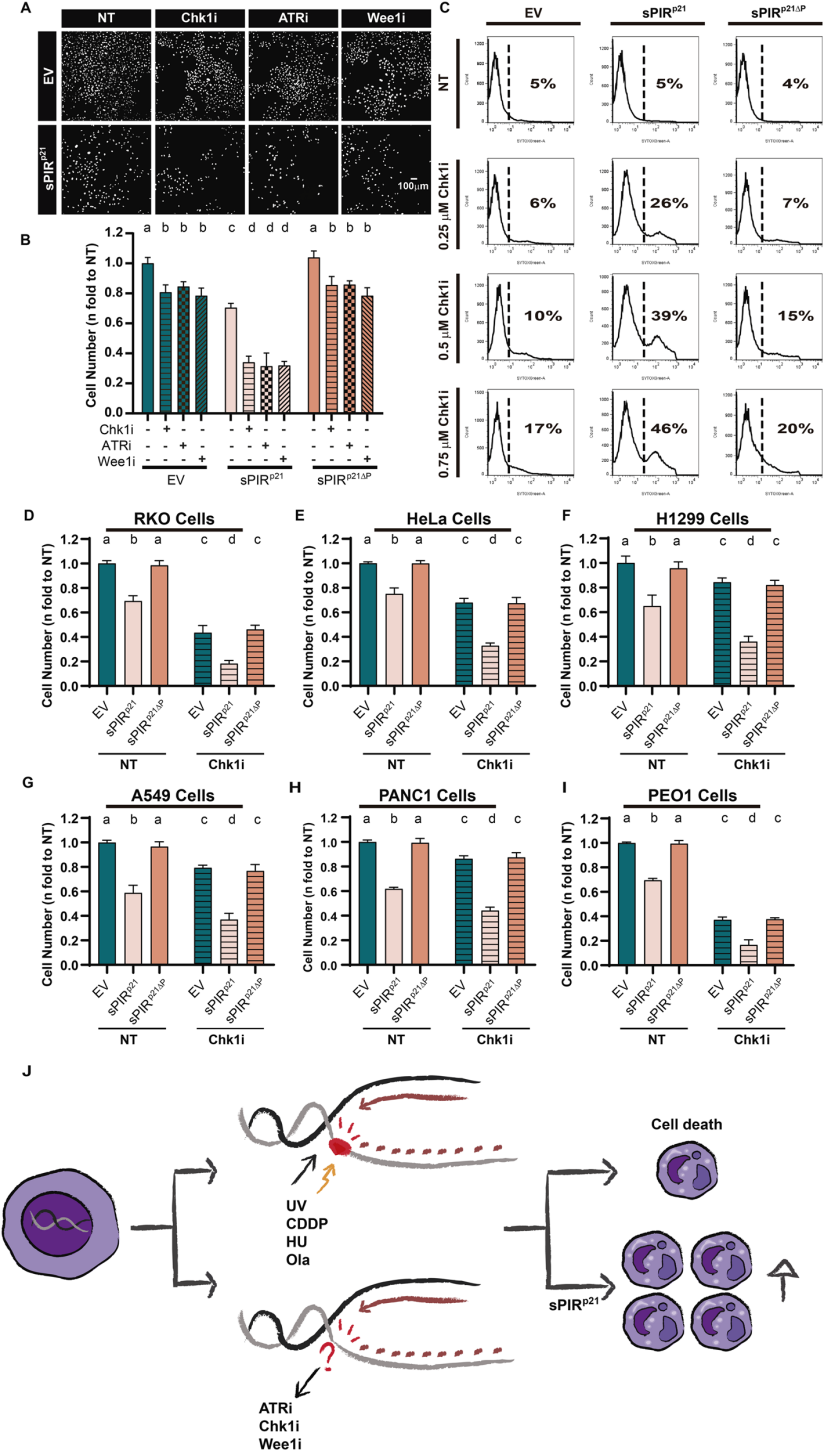
sPIR^p21^ sensitizes cells to agents that alter the replication dynamics without directly generating DNA damage. **A** Representative images of U2OS cells transduced with EV or sPIR^p21^ particles, mock treated (NT) or treated with Chk1i (Gö6976, 0.25 µM), ATRi (VE821, 1.5 µM), Wee1i (MK1775, 150 nM). 6 days after treatment, cells were fixed and stained with DAPI. **B** Cell number (mean ± SD) relative to EV-transduced, untreated U2OS cells. The total cell number in 3 wells from a 96-well plate was counted for each condition. *N* = 3 (Statistics: one-way ANOVA, Tukey post test). **C** Flow cytometry detection of SYTOX Green stained U2OS cells transduced with EV, sPIR^p21^ or sPIR^p21ΔP^ particles, mock treated (NT) or treated with Chk1i at the indicated doses. The dotted line represents the separation between the cells that stain positive or negative for SYTOX Green. On the right side of the dotted line, the numbers indicate the percentages of the cell population displaying high intensity of SYTOX green. A representative experiment is shown. *N* = 3. **D** Cell number (mean ± SD) relative to the untreated RKO sample transduced with EV. 6 days after Chk1i (1.5 μM) treatment, the total cell number in 3 wells from a 96-well plate was counted for each condition. *N* = 3 (Statistics: one-way ANOVA, Tukey post test). **E** Cell number (mean ± SD) relative to the untreated HeLa sample transduced with EV. 6 days after Chk1i (1.5 μM) treatment, the total cell number in 3 wells from a 96-well plate was counted for each condition. *N* = 3 (Statistics: one-way ANOVA, Tukey post test). **F** Cell number (mean ± SD) relative to the untreated H1299 sample transduced with EV. 6 days after Chk1i (1.5 μM) treatment, the total cell number in 3 wells from a 96-well plate was counted for each condition. *N* = 3 (Statistics: one-way ANOVA, Tukey post test). **G** Cell number (mean ± SD) relative to the untreated A549 sample transduced with EV. 6 days after Chk1i (2.5 μM) treatment, the total cell number in 3 wells from a 96-well plate was counted for each condition. *N* = 3 (Statistics: one-way ANOVA, Tukey post test). **H** Cell number (mean ± SD) relative to the untreated PANC1 sample transduced with EV. 6 days after Chk1i (3 μM) treatment, the total cell number in 3 wells from a 96-well plate was counted for each condition. *N* = 3 (Statistics: one-way ANOVA, Tukey post test). **I** Cell number (mean ± SD) relative to the untreated PEO1 sample transduced with EV. 6 days after Chk1i (2.5 μM) treatment, the total cell number in 3 wells from a 96-well plate was counted for each condition. *N* = 3 (Statistics: one-way ANOVA, Tukey post test). **J** Model summarizing the versatility of p21: the cell death triggered by inducers of DNA damage or inhibitors of checkpoint proteins (which do not cause direct DNA damage) is enhanced by expression of sPIR^p21^.

We then wondered if sPIR^p21^ also amplifies the cell killing potential of agents with a mechanism of action that does not solely rely on the accumulation of replication barriers. Since tumor cells are under constant oncogenic stress, they become addicted to checkpoint signals arising from kinases such as ATR (Ataxia Telangiectasia and Rad3-related protein) [35, 36], Chk1 (checkpoint kinase 1) [37] and Wee1 [38, 39]. Inhibitors of these kinases are therefore promising agents for cancer therapy and are currently being evaluated in clinical trials [40]. Given the potential relevance of such agents in the clinic and the fact that their killing potential depends on dysregulated origin firing, a variable that is, at least partially, dissociated from the accumulation of replication barriers, we tested the effect of sPIR^p21^ on the killing potential of ATR, Chk1 and Wee1 inhibitors. As shown in Fig. 5A, B, sPIR^p21^, but not sPIR^p21ΔP^, further reduces the cell number after ATR, Chk1 and Wee1 inhibition. Interestingly, the reduction in the cell number that resulted from the combination of Chk1 inhibition and sPIR^p21^ expression correlated with a bona fide augmentation of cell killing (Fig. 5C). Such a potentiating effect of sPIR^p21^ on the cell killing abilities of a Chk1 inhibitor was observed in different tumor cell lines (Fig. 5D–I and Supplementary Fig. 4A–G). Remarkably, in all cases there is no effect of sPIR^p21^ on the S phase transit before treatment, therefore discarding a potential contribution of an sPIR^p21^-mediated arrest to the reduction in cell number (Supplementary Fig. 4H, I). These results lead us to the conclusion that the sPIR^p21^ is an enhancer of cell killing after exposure to DNA damaging agents (UV, CDDP, HU, Ola) but also in combination with checkpoint inhibition (Fig. 5J) which highlights its versatility at amplifying the cytotoxic effect of chemotoxins with different mechanisms of action.

**Fig. 6:**
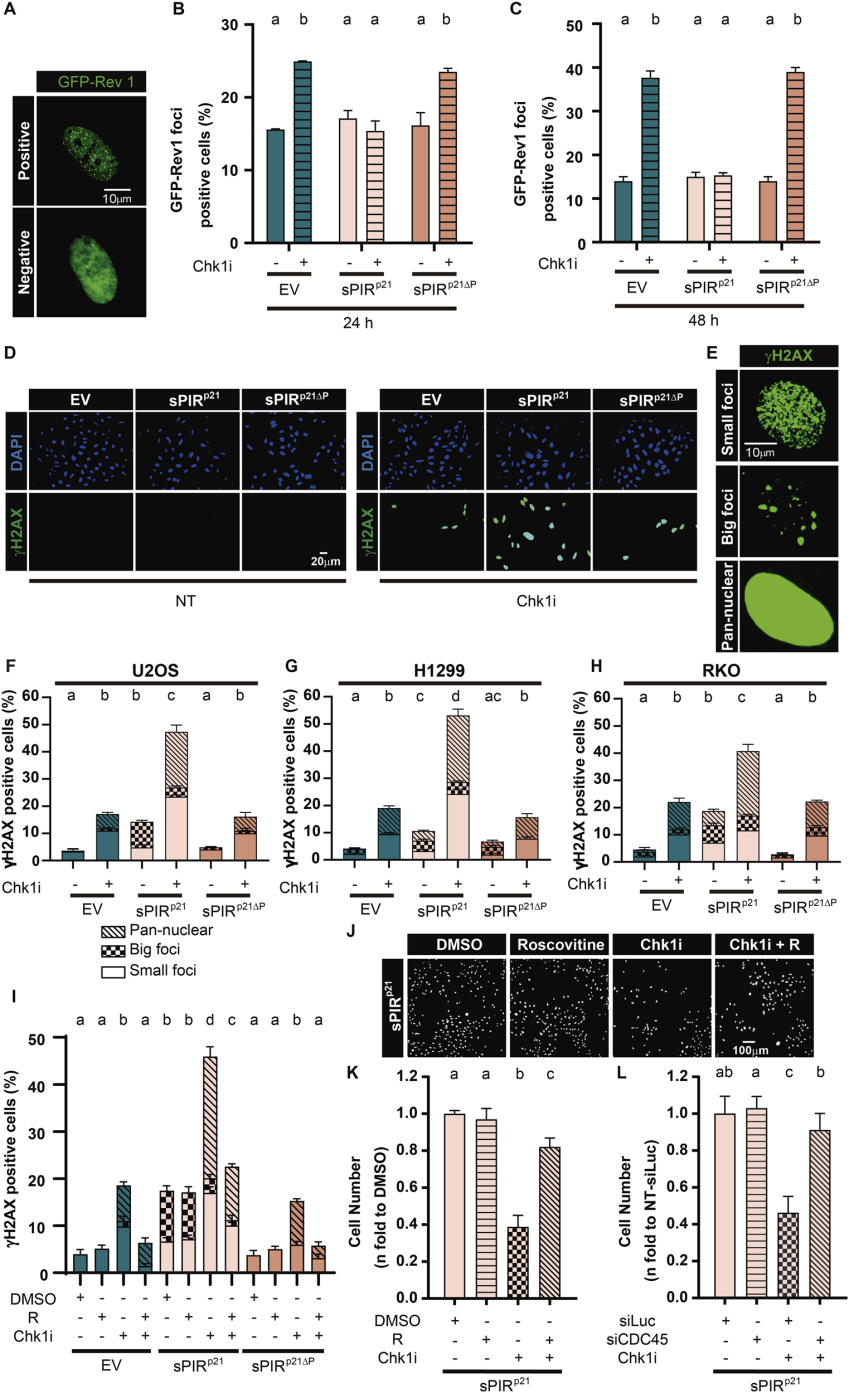
Expression of sPIR^p21^ potentiates the mechanism of cell killing previously reported for Chk1 inhibition. **A** Representative images of GFP-Rev1 transfected U2OS cells. A positive and negative nucleus with and without focal organization of GFP-Rev1 is shown. **B** Percentage (mean ± SD) of U2OS cells with nuclear foci of GFP-Rev1. Samples were transfected with GFP-Rev1 and 5 h later transduced with EV, sPIR^p21^ or sPIR^p21ΔP^. 48 h later, cells were treated with Chk1i (0.5 µM) or DMSO and fixed 8 h after treatment. At least 200 nuclei positive for both GFP and myc/sample were quantified. *N* = 3 (Statistics: one-way ANOVA, Tukey post test). **C** Percentage (mean ± SD) of U2OS cells with nuclear foci of GFP-Rev1. Samples were transfected with GFP-Rev1 and 5 h later transduced with EV, sPIR^p21^ or sPIR^p21ΔP^. After 48 h, cells were treated with Chk1i (0.5 µM) or DMSO and fixed 24 h after treatment. 200 nuclei positive for both GFP and myc were analyzed/sample. *N* = 3 (Statistics: one-way ANOVA, Tukey post test). **D** Representative images showing pan-nuclear γH2AX induction in U2OS cells transduced with the indicated particles after mock treatment (NT) or Chk1i treatment (0.5 µM). **E** Representative images showing different nuclear organization patterns of γH2AX in U2OS cells. **F** Percentage (mean ± SD) of U2OS cells positive for pan-nuclear, small or big γH2AX focal organization. Samples were transduced as indicated in the panel and 24 h later treated with Chk1i (0.5 µM) and fixed 24 h after treatment. At least 200 cells/sample were analyzed. *N* = 3 (Statistics: one-way ANOVA, Tukey post test). **G** Percentage (mean ± SD) of H1299 cells positive for pan-nuclear, small or big γH2AX focal organization. Samples were transduced as indicated in the panel and 24 h later treated with Chk1i treatment (1 µM) and fixed 24 h after treatment. At least 200 cells/sample were analyzed. *N* = 3 (Statistics: one-way ANOVA, Tukey post test). **H** Percentage (mean ± SD) of RKO cells positive for pan-nuclear, small or big γH2AX focal organization. Samples were transduced as indicated in the panel and 24 h later treated with Chk1i treatment (1 µM) and fixed 24 h after treatment. At least 200 cells/sample were analyzed. *N* = 3 (Statistics: one-way ANOVA, Tukey post test). **I** Percentage (mean ± SD) of U2OS cells positive for pan-nuclear, small or big γH2AX focal organization. Samples were transduced as indicated in the panel and 24 h later treated with Chk1i (0.5 µM), roscovitine (2.5 µM) or both, and fixed 24 h later. At least 200 cells/sample were analyzed. *N* = 3 (Statistics: one-way ANOVA, Tukey post test). **J** Representative images of DAPI-stained nuclei from U2OS cells transduced with sPIR^p21^ and 48 h later treated with DMSO, or with Chk1i (0.5 µM), roscovitine (2.5 µM) or both for 6 days. **K** Cell number (mean ± SD) of experiment shown in (**J**). Each condition was expressed relative to the DMSO control. The total cell number in 3 wells from a 96-well plate was counted for each condition. *N* = 3 (Statistics: one-way ANOVA, Tukey post test). **L** Cell number (mean ± SD) relative to untreated (NT) siLuc-transfected U2OS cells. Samples were transfected with siLuc or siCDC45 and 5 h later transduced with sPIR^p21^. 24 h later samples were mock treated (NT) or treated with Chk1i (0.5 µM). 6 days later, the total cell number in 3 wells from a 96-well plate was counted for each condition. *N* = 3 (Statistics: one-way ANOVA, Tukey post test).

We explored the mechanism by which the sPIR^p21^ potentiates the cell killing caused by Chk1 inhibition. The mechanism of cell killing triggered by checkpoint inhibitors is associated with dysregulated origin firing and CDK2 hyperactivation. In principle, such a mechanism does not involve TLS, but lately it has been reported that the TLS inhibitor JH-RE-06, which inhibits Rev1/Pol ζ-dependent TLS, augments the cell killing potential of ATR and Wee1 inhibitors [41]. In agreement with that report, we found an increase in the number of cells with Rev1 foci after Chk1 inhibition in control and sPIR^p21ΔP^ but not sPIR^p21^ expressing samples (Fig. 6A–C), suggesting that the sPIR^p21^ impairs replication events also after Chk1 inhibition. Cell killing by Chk1 inhibition is preceded by the accumulation of cells revealing high levels of replication stress (an intense and pan-nuclear γH2AX distribution) which has been associated with a commitment to cell death [42, 43]. The proportion of cells with pan-nuclear γH2AX steeply increases in samples treated with Chk1i expressing sPIR^p21^, but not in those expressing sPIR^p21ΔP^ (Fig. 6D–H). The accumulation of pan-nuclear γH2AX after Chk1i or Chk1 KD has been associated with the excess CDK2 activity resulting from the loss of its Chk1-mediated inhibition [44, 45]. Accordingly, the increased pan-nuclear γH2AX caused by sPIR^p21^ in Chk1i treated samples was fully prevented when using a CDK2 inhibitor, roscovitine (Fig. 6I). This result suggests that sPIR^p21^ enhances the replication stress caused by dysregulated CDK2 activity in Chk1 inhibited cells. Roscovitine treatment also counteracts the cell killing activities of Chk1i [46, 47]. Such an effect is also recapitulated by the depletion of CDC45, a replication origin associated factor that reverts the profound alterations in DNA replication caused by Chk1i [47]. We confirmed those observations in control samples without peptide expression or with expression of sPIR^p21ΔP^ (Supplementary Fig. 5) and determined that the potentiation of cell killing by the Chk1i by co-treatment with sPIR^p21^ was attenuated when treating cells with roscovitine or downregulating CDC45 (Fig. 6J–L and Supplementary Fig. 5). Together, these results demonstrate that the mechanism of action of Chk1i is not modified by the expression of sPIR^p21^ but instead, that sPIR^p21^ augments the efficiency of the Chk1i-mediated cell killing at multiple levels involving at least the reduction of Rev1-mediated TLS and also, the potentiation of CDK2 hyperactivation-dependent events.

**Fig. 7:**
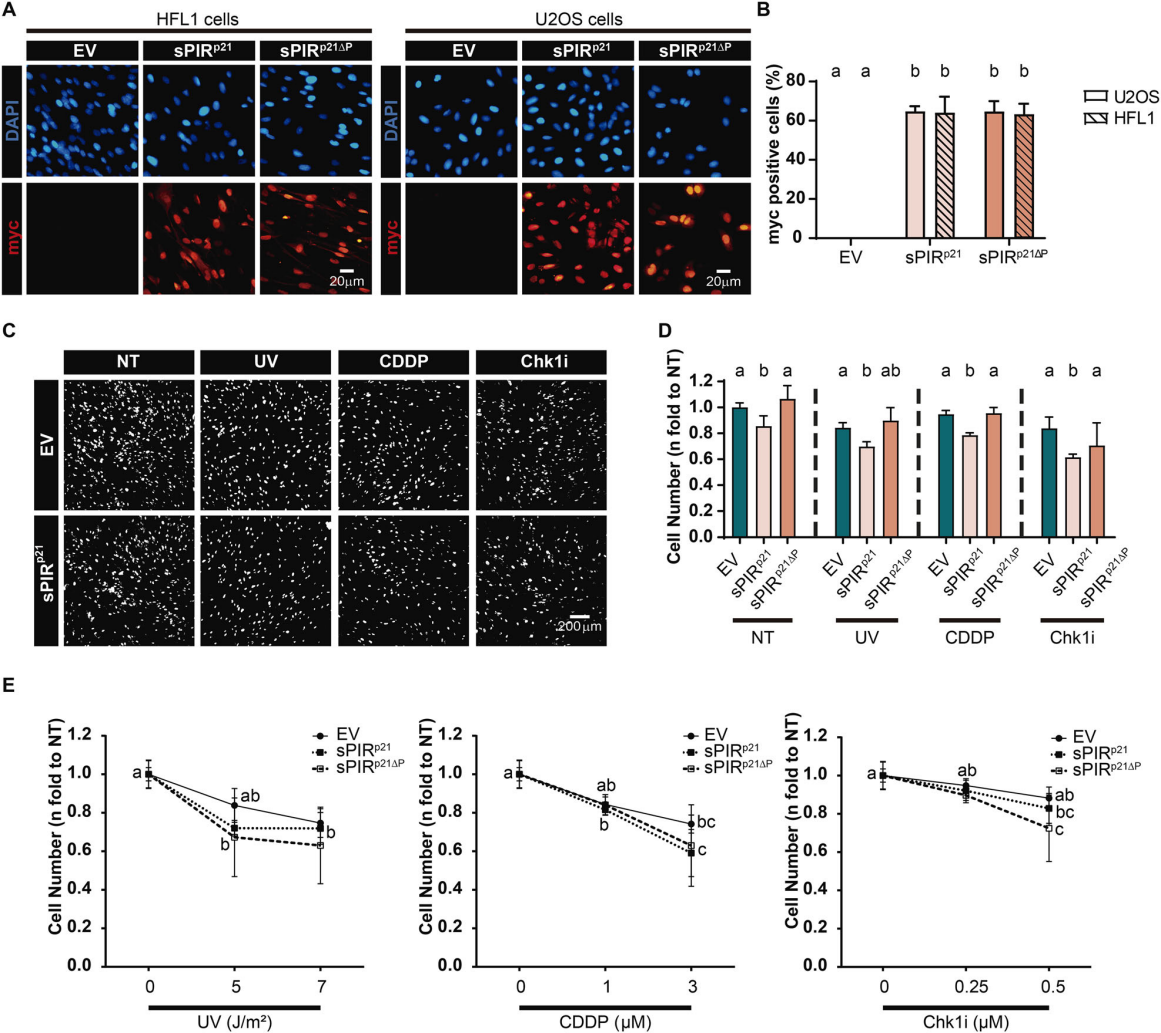
Expression of sPIR^p21^ does not potentiate the mechanism of cell killing by UV, CDDP and Chk1 inhibition in non-cancerous HFL1 cells. **A** Representative images of HFL1 (left panel) and U2OS (right panel) cells transduced with the indicated vectors. Detection of p21 variants was performed by immunofluorescence using a specific myc-tag antibody. **B** Percentage (mean ± SD) of cells expressing the indicated p21 mutants. U2OS and HFL1 cells were transduced and fixed 48 h later. At least 500 nuclei/sample were analyzed. *N* = 2 (Statistics: one-way ANOVA, Tukey post test). **C** Representative images of DAPI-stained HFL1 cells transduced with either EV or sPIR^p21^ particles. 48 h later, cells were either mock treated (NT) or treated with UV (5 J/m^2^), CDDP (1 μM) or Chk1i (Gö6976 0.25 µM) and fixed 6 days later. **D** Cell number (mean ± SD) expressed as fold changes with respect to the EV transduced, mock treated (NT) HFL1 cells shown in (**C**). The total cell number in 3 wells from a 96-well plate was quantified for each condition. *N* = 2 (Statistics: one-way ANOVA, Tukey post test, samples were compared within each treatment). **E** Cell number (mean ± SD) relative to untreated HFL1 cells transduced with either EV, sPIR^p21^ or sPIR^p21ΔP^. 6 days after the treatment with the indicated UV, CDDP or Chk1i doses, the total cell number in 3 wells from a 96-well plate was counted for each condition. *N* = 2 (Statistics: one-way ANOVA, Tukey post test). The same source data used in **D** was used here.

Finally, we tested the effect of sPIR^p21^ on a non-cancer cell line. We used HFL1 fibroblasts, which were transduced with an efficiency similar to U2OS (Fig. 7A, B). Having demonstrated that sPIR^p21^ expression was sufficient, we found that sPIR^p21^ caused a reduction in the number of untreated cells (Fig. 7C, D) which recapitulated the effect on cancer cells (Fig. 2A, B). The cytotoxic effect of chemotoxins was detected in HFL1 cells (Fig. 7D), hence demonstrating that they were also working in this setting. However, in contrast to cancer cells, there was no evidence of synergy between sPIR^p21^ and the cytotoxic agents (Fig. 7D, E), hence indicating that sPIR^p21^ selectively affected cancer cells.

### The PIR of p21 is not unique in its ability to enhance the cell killing by genotoxins

**Fig. 8:**
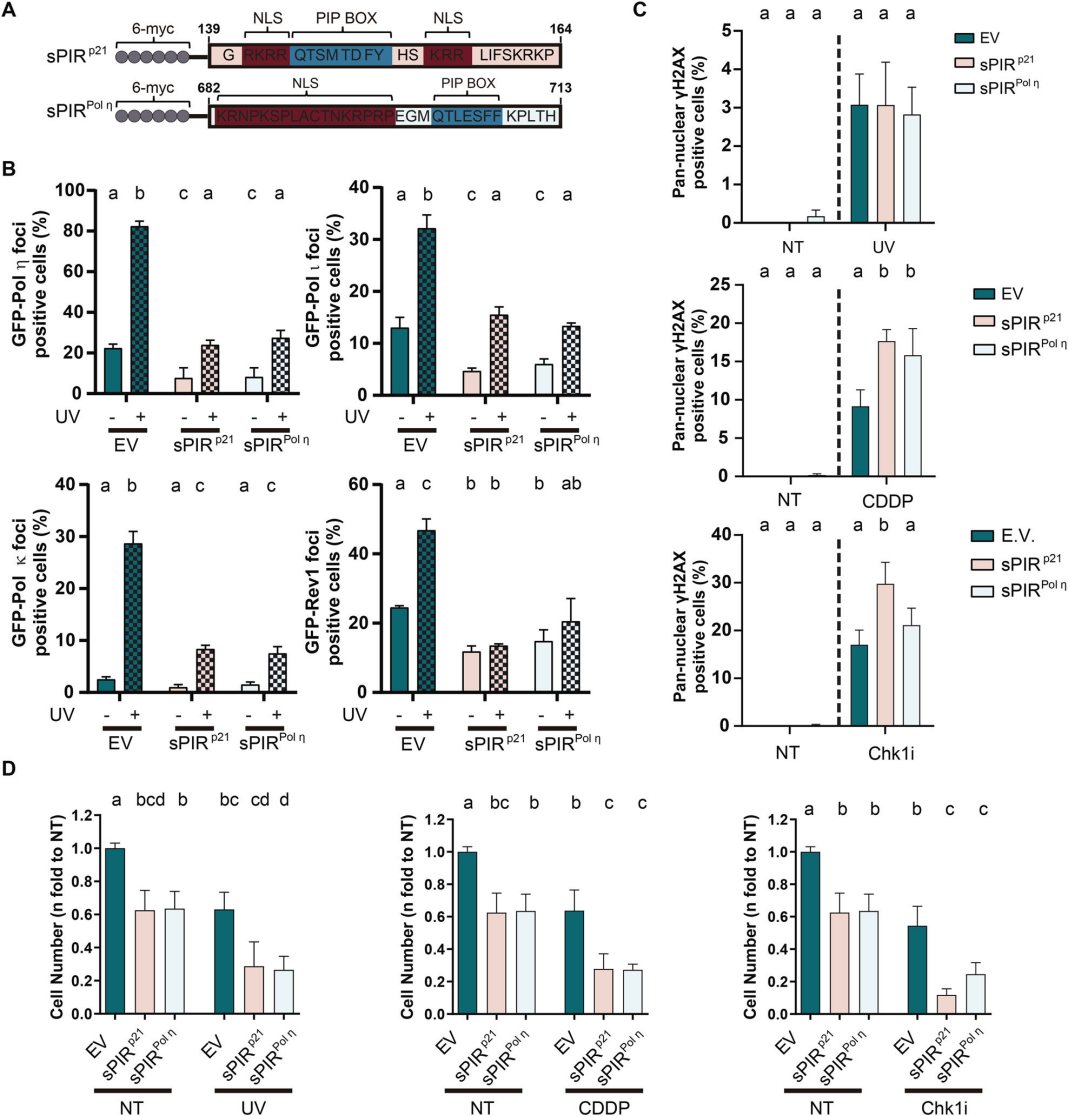
sPIR^Pol η^ is as effective as sPIR^p21^ in enhancing the killing potential of agents that trigger a DNA damage response. **A** On top: Representative image of sPIR^p21^ peptide. Below: Representative image of sPIR^Pol η^ peptide consisting of amino acid residues 682 to 713 derived from Pol ƞ, fused to a 6-myc-tag at the N-terminus. The 7-amino acid sequence corresponding to the PIP box is indicated in blue. The NLS is represented in dark red. NLS: nuclear localization signal. For further details about the variants used, see “Materials and methods” section. **B** Percentage (mean ± SD) of U2OS cells with nuclear foci of the indicated polymerase. Samples were transfected with the indicated GFP-Pol and 5 h later transduced with EV, sPIR^p21^ or sPIR^Pol η^ viral particles. 48 h later, cells were irradiated with UV (40 J/m^2^) and fixed after 4 h. Transduced cells were identified by a myc-tag specific antibody, whereas GFP-focal organization was determined by monitoring GFP. Between 100–200 nuclei positive for both GFP and p21/sample were analyzed. *N* = 3 (Statistics: one-way ANOVA, Tukey post test). **C** Percentage (mean ± SD) of U2OS cells positive for pan-nuclear γH2AX transduced with indicated vectors. Cells were fixed 24 h after Chk1i (0.5 µM), UV (5 J/m^2^) or CDDP (6 μg/ml) treatment. At least 300 cells/sample were analyzed. *N* = 3 (Statistics: One-way ANOVA, Tukey post test, samples were compared within each treatment). **D** Cell number (mean ± SD) relative to EV untreated control for U2OS cells transduced with the indicated vectors, mock treated (NT) or treated with UV (5 J/m^2^), CDDP (1 μM) or Chk1i (Gö6976, 0.25 µM) and fixed 6 days later. The total cell number in 3 wells from a 96-well plate was counted for each condition. *N* = 3. NT samples are the same for all three plots (Statistics: one-way ANOVA, Tukey post test).

Because sPIR^p21^ is specialized in S-Pol displacement from replication factories, we wondered if the PIR domain from an S-Pol would suffice to enforce such displacement and to enhance the cell killing of different genotoxic agents. We generated a construct encoding the 32 C-terminal amino acid residues of Pol eta (sPIR^Pol η^) (Fig. 8A). sPIR^Pol η^ was as efficient as sPIR^p21^ at inhibiting Pol η foci formation, hence indicating that Pol η can be displaced from replication factories by an excess of its own PIR^Pol η^ (Fig. 8B). Intriguingly, the overexpression of sPIR^Pol η^ also displaced the other S-Pols to the same extent as sPIR^p21^ (Fig. 8B). Such a similarity in the mechanism of action of sPIR^Pol η^ and sPIR^p21^ is also observed at the levels of the disruption of Pol η:PCNA interaction (Supplementary Fig. 6A, B). When comparing sPIR^Pol η^ with sPIR^Pol ηΔP^, a mutant with a disrupted PCNA binding domain with a similar transduction efficiency (Supplementary Fig. 7A, B) we found that sPIR^Pol η^ is able to interact with PCNA while the sPIR^Pol ηΔP^ is not (Supplementary Fig. 7C, D). Hence, the efficient interaction of sPIRs with PCNA and the displacement of S-Pols from PCNA is not exclusive to the strongest PIR, that of p21. We then compared the sPIR^Pol η^ and the sPIR^p21^ effects in γH2AX and cell survival assays after UV, CDDP and Chk1 inhibition. Also in these experiments, the effects of sPIR^p21^ and sPIR^Pol η^ were indistinguishable (Fig. 8C, D), and dependent on PCNA binding (Supplementary Fig. 7E, F) with the exception of an undetectable effect of sPIR^Pol η^ in combination with Chk1i on the percentage of cells with pan-nuclear γH2AX despite comparable killing potency (Fig. 8C, D). Collectively, these results suggest that PIR domains other than the one in p21 have a similar capacity to disrupt PCNA interactions with S-Pols, triggering the versatile enhancement of cell killing by genotoxic agents.

## Discussion

Here we describe a peptide-based adjuvant reagent with potential to enhance the cell killing by several chemotherapeutic agents characterized by dissimilar modes of action. Such versatility of the PIR peptides is achieved in S phase, specifically by enhancing the replication stress caused by the treatment, in particular, at the level of preventing the participation of the four S-Pols in the DNA damage response to chemotoxins. We observed that the attenuation of the cellular capability to tolerate chemotherapy achieved by the PIR peptides is fully dependent on their PCNA binding capacity, independently of the strength of their binding reported in in vitro assays. Hence, the excess amount of molecules occupying the interdomain connecting loop (IDCL) of PCNA rather than the strength of the interaction of the PIR peptides with such a region of PCNA may be crucial to causing unsuccessful completion of S-Pols-mediated DNA replication in S phase, which is detrimental to cell survival independently of the DNA damaging agent used.

### sPIR^p21^, a prime example of the principle of potentiating chemotoxins via peptides targeting the IDCL of PCNA

Displacement of PCNA interaction partners such as S-Pols is most likely achieved when higher affinity PIP boxes are used for peptide design. PIP boxes that were previously reported to displace Pol η from PCNA are the one of the licensing factor CDT1 and that of methyltransferase Set8 [48]. These proteins share a common feature: high affinity for PCNA [26] and a degron sequence that promotes their degradation when bound to PCNA [25]. Other PIP containing proteins such as FEN1 (Flap Endonuclease 1) or p15, with lower affinity for PCNA and no degron sequence, have no effect on Pol η focal organization [48]. These observations lead to the suggestion that only PIP domains including a degron are able to disrupt the interactions between S-Pols and PCNA [48]. In line with these observations, our results show that the PIR-degron sPIR^p21^ is sufficient to displace S-Pols from PCNA and to increase the cell killing potential of a variety of chemotherapeutic drugs. However, we did not find any direct contribution of the degron to this effect of sPIR^p21^ or full-length sp21. Moreover, overexpression of another small peptide, namely sPIR^Pol η^, which encompasses a PIP box with no degron sequence and intermediate binding affinity towards PCNA [26, 49], displaces S-Pols from PCNA and increases the cell killing potential of a variety of genotoxic agents to a level similarly reached by sPIR^p21^. These results suggest that several PIP motifs, when expressed in isolation, could efficiently impair TLS events, regardless of the degron sequence, interfering with cell growth and exacerbating cell killing when combined with chemotherapeutic drugs. In addition, our results are in line with other reports that demonstrate that exploiting disassembly of the PCNA interactome at partially overlapping but also distinct regions of PCNA can enhance the cell killing potential of chemotherapeutic drugs [50–52]. In summary, the use of small molecules or specific peptides that disrupt the crosstalk of PCNA with its more than 400 protein partners may represent a valid and versatile strategy to enhance current chemotherapeutic strategies.

### Modification of the PCNA interactions by sPIR^p21^ may not be limited to the displacement of S-Pols

Disruption of S-Pol foci in cells treated with DNA damaging agents is considered a marker of TLS inactivation [1]. Remarkably, here we have shown that a small 26-residue peptide covering the C-terminus of p21 achieves the same effect as full-length sp21 in side-by-side experiments. Importantly, such an effect is fully dependent on the ability of this sPIR^p21^ peptide to bind PCNA, as the sPIR^p21ΔP^ mutant completely loses this function. Another line of evidence supporting TLS inhibition by sPIR^p21^ is revealed by the length of nascent DNA tracks that are equally affected by sPIR^p21^ and sp21. In conclusion, the 26 amino acid residues of PIR^p21^ contain all the properties necessary to strongly impair TLS. In addition, experiments performed under unperturbed conditions reveal that sPIR^p21^ affects processes other than TLS, namely: frequency of origin firing, persistent accumulation of damaged DNA in S phase (big γH2AX foci), mitotic index and accumulation of replication-derived defects in G1 (53BP1 nuclear bodies). These features could all be derived from defects generated in or outside S phase, but are unlikely to be directly coupled with the displacement of S-Pols. In particular, literature reports did not find an association between origin firing and TLS [53]. Moreover, all the above-mentioned defects were not observed when expressing sp21, which is an equally effective inhibitor of TLS as sPIR^p21^.

When focusing on DNA damaging treatment conditions, there is also evidence indicating that sPIR^p21^ may target processes other than TLS. While the synergistic effects with CDDP, UV, HU and even Ola could be attributable to the inhibition of TLS, augmentation of cell killing by Chk1i, ATRi or Wee1i is unlikely solely associated to TLS failure. For example, Pol η is known to be inactivated after Chk1 downregulation, therefore does not participate in DNA elongation events after Chk1 downregulation [47], downplaying the potential role of TLS in the DDR that takes place post-Chk1 depletion. Moreover, killing cells by Chk1 inhibition and downstream CDK2 hyperactivation depends, at least partially, on dysregulation of origin firing, as it is prevented if CDK2 is attenuated by roscovitine or if its target CDC45 is depleted [45–47]. In our study, exacerbating the cell killing potential of Chk1 inhibition by sPIR^p21^ was indeed prevented by roscovitine treatment and CDC45 downregulation, which is why we concluded that this capacity of sPIR^p21^ is at least partially independent of TLS inhibition. Together, these data suggest that multiple PCNA functions and interactions are affected by sPIR^p21^ not only at ongoing forks but also in other replication-relevant structures such as origins (this is also supported by the effect on origin firing frequency observed after sPIR^p21^ expression in untreated cells). This could relate to the diversity of PCNA partners that include strategic components of origin firing complexes such as Cdt1, a protein involved in origin licensing, which also harbors a PIR [54]. Together, we believe that separating sPIR^p21^ from other p21 regions permits sPIR’s localization to structures other than the ongoing replisome, a scenario that promotes its interference with PCNA-dependent processes other than TLS, which contributes to the versatility of cell death induction by PIR.

### Displacement of TLS polymerases from PCNA: a powerful strategy to enhance cancer cell killing

So far, the most advanced TLS inhibitors are JH-RE-06 and T2AA. The former targets the interaction of Rev1 with the RIR (Rev1 interacting region) of other polymerases, precluding the interaction of Pol ζ with Rev1, diminishing the global efficacy of the mutagenic TLS dependent on this S-Pol [55, 56]. The latter associates with the IDCL of PCNA precluding the binding of DNA polymerases at the same site also targeted by the PIR peptides used in this study [57]. Both small molecule inhibitors have been reported to enhance the potency of CDDP. JH-RE-06 impairs nascent DNA elongation after CDDP treatment in a manner that is presumably related to the inhibition of S-Pols recruitment to replication factories. However, inhibiting access to the RIR should only partially prevent TLS events, because they could still take place via direct recruitment of S-Pols to the IDCL, the target of T2AA and PIR peptides. T2AA has been shown to reduce TLS events upon DNA damage, underscoring the importance of this interaction site for S-Pol activity. However, T2AA also has a negative effect on BrdU incorporation independently of DNA damage [57]. This indicates that T2AA interferes with R-Pols causing a cell cycle arrest that could protect rather than sensitize cancer cells to genotoxic agents such as CDDP, that cause DNA replication-dependent cell killing. It is therefore significant that sPIR^p21^ does not affect the number of cells entering S phase or the intensity of global DNA synthesis within S phase. This observation recapitulates results obtained with full-length sp21 which prevents the recruitment of S-Pols but not of Pol δ to PCNA [21]. Hence, sPIR^p21^ selectively enforces S-Pol displacement from PCNA. It is unclear why sPIR^p21^ only targets S-Pols despite overexpression with no effect on normal S phase. We speculate that such an effect is associated with the fact that R-Pols are multimeric enzymes with more than one PIR while S-Pols have only one subunit. It is therefore likely that, as demonstrated for other proteins [58], the ability of R-Pols to achieve multiple docking on PCNA may prevail in the presence of excess levels of a single-docking sPIR^p21^ peptide while single-docking S-Pols fail to do so. Importantly also, peptide-based drugs as compared to small molecules have been developed precisely because of their higher target specificities and potencies typically resulting in fewer side effects [59]. Regardless of the mechanism, we believe that the scenario generated by sPIR^p21^ in terms of selectively affecting S phase progression after DNA damage is advantageous when attempting to selectively kill cancer cells. Moreover, while the versatility of JH-RE-06 and T2AA is awaiting to be explored, potentially non-overlapping effects achieved by these reagents suggest that there is room for positive interactions between PIR peptides and chemical TLS inhibitors when combining them with genotoxic drugs.

## Materials and methods

### Cell culture and reagents

U2OS (ATCC) (RRID: CVCL_0042), RKO (ATCC), HeLa (ATCC), H1299 (ATCC), A549 (ATCC), PANC1 (ATCC), and PEO1 (gift from Francis Crick Institute, Baltimore, London, UK) were grown in Dulbecco’s modified Eagle’s medium (Invitrogen) with 10% fetal calf serum (Natocor). HFL1 (ATCC) cells were grown in 50% Dulbecco’s modified Eagle’s medium (Invitrogen) and 50% Ham’s F-12 nutrient mixture medium (Thermo Fisher Scientific), complemented with glutamine (final concentration 2 mM) and 10% fetal calf serum (Natocor). Cells were treated with UV-C irradiation as described previously [22]. CDDP and HU were from Sigma #P4394 and #127-07-1, respectively. Checkpoint Inhibitors Gö6976 (Chk1i), VE821 (ATRi) and MK1775 (Wee1i) were purchased from Tocris #2253, Adooq Bioscience #A11605 and Selleckchem #S1525 respectively. Ola was acquired from Selleckchem #S1060. All cell lines were kept in a humidified, 5% CO_2_ incubator and passaged as needed. Cell lines were periodically checked for mycoplasma contamination. None of the cell lines used in this study are listed as of commonly misidentified cell lines reported by the International Cell Line Authentication Committee.

### siRNAs, vector expression plasmids and lentiviral infection

Transfection of siRNAs and plasmid expression vectors were conducted using jetPRIME (Polyplus) following the manufacturer’s instructions. Except for the experiments that evaluated survival capacity, cells were harvested 48 h or 72 h after transfection. GFP-Pol η and GFP-Pol κ were gifts from Dr. A. Lehmann, (Genome Damage and Stability Centre, School of Life Sciences, University of Sussex, Falmer, Brighton BN1 9RQ, UK) [60]; GFP-Pol ι was donated by Dr. R. Woodgate (Laboratory of Genomic Integrity, National Institute of Child Health and Human Development, National Institutes of Health, Bethesda, MD 20892-3371, USA) and GFP-Rev1 was provided by Dr. E. Friedberg (Department of Pathology, UT Southwestern Medical Center, Dallas, TX 75390, USA). GFP-PCNA was kindly given to us by Dr. M C Cardoso (Max Delbruck Center for Molecular Medicine, Berlin, Germany) [61].

siRNAs were purchased from Dharmacon, Eurofins Genomics and Qiagen:

siLuc: 5′-CGUACGCGGAAUACUUCGA-3′ [62];

siCDC45: 5′-GCAAGACAAGAUCACUCAA-3′.

In this work, we used or modified the following expression vectors: CS2MT-EV (The CS2MT vector, which expresses the 6-myc-tag, was kindly provided by Dr. Dave Turner (University of Michigan, MI, USA)), p21 (CS2-p21 (CDK-)), sp21 (CS2MT-p21 (CDK-)) [21, 23], p21ΔP (CS2-p21 (CDK-/PCNA-)) [23], or sp21ΔP (CS2MT-p21 (CDK-/PCNA-)) [23], as previously generated by us.

To generate p21ΔDeg and sp21ΔDeg mutants, p21 (CS2-p21 (CDK-)) and sp21 (CS2MT-p21 (CDK-)) expression vectors were used respectively. Quick-change site-directed mutagenesis (Stratagene) was used. The primers used to generate the degron mutation (R155A) were the following:

Forward: 5′-CTACCACTCCAAAGCCCGGCTGATCTTCTCCAAGAGGAAGC-3′

Reverse: 5′-GCTTCCTCTTGGAGAAGATCAGCCGGGCTTTGGAGTGGTAG-3′

To generate sPIR^p21^ and sPIR^p21ΔP^ mutants, sp21 (CS2MT-p21 (CDK-)) and sp21ΔP (CS2MT-p21 (CDK-/PCNA-)) expression vectors were used, respectively. A PCR product encoding the PCNA-interacting region (PIR) of p21 (amino acid residues 139-164) was obtained, flanked by restriction sites for the enzymes EcoRI and XbaI. The primers used were the following:

Forward: 5′-CGCGAATTCAGGTCGAAAACGGGCGGCAGACC-3′

Reverse: 5′-GCTCTAGATTAGGGCTTCCTCTTGGAGAA-3′

The sites for EcoRI and XbaI were used to insert the PCR fragment into a CS2MT vector, immediately after the last myc of the 6-myc-tag in the vector.

sp21, sPIR^p21^ and sPIR^p21ΔP^ mutants were subcloned from the CS2MT expression vectors into the lentiviral transfer vector pLenti CMV/TO Puro (Plasmid #17482, Addgene, 3rd generation), using the restriction enzymes BamHI and XbaI.

To generate sPIR^Pol η^ peptide expressing plasmid, sequences coding for the 6-myc-tag and PIR^Pol ƞ^ were fused from sPIR^p21^ and GFP-Pol ƞ lentiviral expression vectors, respectively, as follows:

A PCR product encoding the PCNA-interacting region (PIR) of Pol ƞ (amino acid residues 682-713) was obtained from GFP-Pol ƞ vector. The primers used were the following:

Forward: 5′-GACTTGACGCGTAAAAGAAATCCCAAGAGCCCT-3′

Reverse: 5′-ACCACTTTGTACAAGAAAGCTGGG-3′

A PCR product encoding the 6-myc-tag was obtained from the sPIR^p21^ lentiviral expression vector. The primers used were the following:

Forward: 5′-GCAGGCTCCACCATGGGAAC-3′

Reverse: 5′-GGGATTTCTTTTACGCGTCAAGTCCTCTTCAGAAATGAGCTTTTGCTCCATGGTGAGGTCG-3′

Then, the fragments obtained in both PCRs were subjected to overlapping PCR (underlined above: regions of overlap in the primers), using the following primers:

Forward: 5′-GCAGGCTCCACCATGGGAAC-3′

Reverse: 5′-ACCACTTTGTACAAGAAAGCTGGG-3′

The restriction enzymes BamHI and XbaI were used to digest the product of the overlapping PCR and the sPIR^p21^ (CMV/TO-sPIR^p21^) lentiviral expression vector. Both digestion products were subjected to ligation.

sPIR^Pol ηΔP^ was obtained by site-directed mutagenesis by Gene Universal using sPIR^Pol η^ as backbone. Point mutations that eliminate its ability to bind PCNA (ΔP: L704A/ F707A/ F708A) that were previously reported [63] were introduced.

Lentiviral particles were obtained by transfecting HEK293T cells with jetPRIME in 60 mm dishes in a 5:5:1 ratio (pLenti: psPAX2:PMD2). Forty-eight hours after transfection, lentiviral particles were collected from the supernatant of HEK293T cells, centrifuged, filtered with a 0.45 μm filter and stored at −80 °C. Lentiviral preparations were slowly thawed and used to infect cells in 24/12/6-well dishes in the presence of 8 µg/ml polybrene.

**Table:** 

Plasmid	Description	Mutations	Effect of mutations
p21	p21 peptide	W49R, F51S, D52A	Prevents CDK binding
p21ΔDeg	p21 peptide	1 W49R, F51S, D52A 2 R155A	1 Prevents CDK binding 2 Prevents PCNA-dependent degradation
sp21	p21 peptide, with a 6-myc-tag at the N-terminus	W49R, F51S, D52A	Prevents CDK binding
sp21ΔDeg	p21 peptide, with a 6-myc-tag at the N-terminus	1 W49R, F51S, D52A 2 R155A	1 Prevents CDK binding 2 Prevents PCNA-dependent degradation
p21ΔP	p21 peptide	1 W49R, F51S, D52A 2 M147A, D149A, F150A	1 Prevents CDK binding 2 Prevents PCNA binding
sp21ΔP	p21 peptide, with a 6-myc-tag at the N-terminus	1 W49R, F51S, D52A 2 M147A, D149A, F150A	1 Prevents CDK binding 2 Prevents PCNA binding
sPIR^p21^	Amino acid residues 139-164 of p21, with a 6-myc-tag at the N-terminus		
sPIR^p21ΔP^	Amino acid residues 139-164 of p21, with a 6-myc-tag at the N-terminus	M147A, D149A, F150A	Prevents PCNA binding
sPIR^Pol η^	Amino acid residues 682-713 of Pol η, with a 6-myc-tag at the N-terminus		
sPIR^Pol ηΔP^	Amino acid residues 682-713 of Pol η, with a 6-myc-tag at the N-terminus	L704A, F707A, F708A	Prevents PCNA binding

### Immunostaining and fluorescence detection

For quantification and immunodetection of specialized DNA polymerases in the Y-family, p21, 53BP1 and γH2AX, cells were fixed in 2%–4% paraformaldehyde (PFA)/2% sucrose and later permeabilized with 0.1% Triton X-100 in phosphate-buffered saline (PBS) as described previously [22, 64]. When using BrdU (10 μM, Sigma Cat# B9285), cells were fixed with methanol-acetone and then subjected to a denaturing step with 1.5 N HCl for 30 min. Such a procedure exposes the BrdU epitopes enabling their detection with specific antibodies. The visualization of 53BP1 nuclear bodies in EdU negative cells, was achieved by pulse-labeling cells with EdU (10 µM) for 15 min before fixation with 4% PFA/2% sucrose. After fixation, samples were permeabilized and the EdU detection was carried out using a commercial kit following the manufacturer’s instructions (Click-iT EdU kit Invitrogen Cat# C10338). Later on, a regular immunofluorescence was performed to reveal 53BP1. For the visualization of γH2AX in EdU positive and negative cells, cells were pulsed with EdU (10 µM) for 15 min before fixation with 4% PFA/2% sucrose. Samples were permeabilized, EdU staining was carried out using EdU detection kit following the manufacturer’s instructions (Click-iT EdU kit Invitrogen Cat# C10338) and later a regular immunofluorescence was performed for γH2AX staining. For the detection of proteins using immunofluorescence technique, cells were initially fixed in 2%–4% paraformaldehyde (PFA)/2% sucrose and permeabilized with 0.1% Triton X-100 in phosphate-buffered saline (PBS). In all cases, blocking was performed overnight in 2% donkey serum (Sigma) in PBS-0.05% Tween before incubation with primary antibodies. Cover slips were then incubated for 1 h with primary antibodies. Primary antibodies used in this study were: anti-53BP1 (Santa Cruz Biotechnology Cat# sc-22760, RRID: AB_2256326), anti-γH2AX (Millipore Cat# 05-636, RRID: AB_309864), anti-BrdU (GE Healthcare Cat# RPN 202, RRID: AB_2314032), anti-p21 (C-19, Santa Cruz Biotechnology Cat# sc-397, RRID: AB_632126), anti-myc (Bethyl Cat# A190-105A, RRID: AB_67390), anti-BrdU (Accurate Chemical and Scientific Corporation Cat# OBT-0030, RRID: AB_2341179 to detect CldU), anti-BrdU (BD Biosciences Cat# 347580, RRID: AB_400326 to detect IdU). Secondary anti-mouse/rabbit-conjugated 488/546 Alexa antibodies were purchased from Invitrogen. Specialized Y polymerases and PCNA, tagged with GFP, were visualized by GFP autofluorescence. Nuclei were stained with DAPI (Sigma-Aldrich). Images were obtained with an LSM 510 META confocal microscope or a Zeiss Axio Observer 3 microscope. When quantifying 53BP1 nuclear bodies in EdU negative cells, all small focal structures of <1.5 µm were not counted, as 53BP1 nuclear bodies were described as 2–3 µm structures, while smaller structures are associated with S phase foci [65]. For the quantification of GFP-tagged specialized Y polymerases foci, the number of foci was determined for each polymerase, setting up an average of foci detected in untreated conditions. After induction of DNA damage, the number of positive nuclei was determined by counting nuclei with more foci than the number determined for the untreated condition. To quantify mitotic index, DAPI (Sigma-Aldrich) staining served to identify cells which were transiting mitosis. When needed, Z-stacks were acquired with a Zeiss LSM 880 confocal microscope. Maximum intensity projections were generated using ImageJ (ImageJ 1.52a) and Black ZEN Imaging software (Zeiss).

### Proximity ligation assay (PLA)

U2OS cells were seeded onto 12-well plates. Twenty-four hours later they were transfected with the indicated plasmids and/or transduced with the indicated lentiviral particles. Twenty-four hours later they were reseeded onto 12 mm diameter coverslips in 24-well plates. Twenty-four hours later, only for GFP:PCNA PLA, the 4-h UV treatment indicated was carried out, followed by pre-extraction with ice cold CSK buffer (10 mM PIPES pH 6.8, 100 mM NaCl, 300 mM sucrose, 3 mM MgCl_2_, 1 mM EGTA, 0.5% Triton X-100) for 2 min. For both PLAs (GFP:PCNA and myc:PCNA), cells were fixed in 4% paraformaldehyde (PFA)/2% sucrose and later on permeabilized with 0.1% Triton X-100 in phosphate-buffered saline (PBS). The primary antibodies used were: anti-PCNA (Santa Cruz Biotechnology, Cat# sc-56, RRID: AB_628110), anti-myc (Bethyl Cat# A190-105A, RRID: AB_67390), and anti-GFP (Thermo Fisher Scientific Cat# G10362, RRID:AB_2536526). Interaction between exogenous 6-myc-tagged peptides and PCNA or exogenous, GFP-tagged Pol ƞ and PCNA was detected following manufacturer’s instructions using Duolink In Situ kits: Duolink In Situ PLA Probe Anti-Mouse PLUS 8.0 (Sigma-Aldrich Cat# DUO92001, RRID:AB_2810939), Duolink In Situ PLA Probe Anti-Rabbit MINUS (Sigma-Aldrich Cat# DUO92005, RRID:AB_2810942) and Duolink In Situ Detection Reagents Red (Sigma-Aldrich Cat# DUO92008). Briefly, PLA is based on detecting proteins in close proximity (30–40 nm) by using different species of primary antibodies. A pair of oligonucleotide-tagged secondary antibodies (PLA probes) detect the primary antibodies bound to the proteins of interest and generate a signal only when the two PLA probes have bound in close proximity, meaning that the target proteins are localized in close proximity. The signal from each detected pair of PLA probes is visualized as an individual fluorescent spot. These PLA signals can be quantified (counted) and assigned to a specific subcellular location based on microscopic images. Images were obtained with an LSM 510 META confocal microscope and PLA foci per nucleus were counted using CellProfiler.

### DNA fiber spreading

DNA fiber spreading was performed exactly as previously described [22, 47, 64]. Briefly, to incorporate thymidine analogs, exponentially growing cells were pulse-labeled with CldU (20 µM) for 10 min, washed twice, and incubated with IdU (200 µM) for additional 30 min. Antibodies used were mouse anti-BrdU (BD Biosciences Cat# 347580, RRID:AB_10015219) to detect IdU, donkey anti-mouse Alexa 546 secondary antibody (Thermo Fisher Scientific Cat# A10036, RRID:AB_11180613), rat anti-BrdU (Accurate Chemical and Scientific Corporation Cat# YSRTMCA2060GA, RRID:AB_2631302) to detect CldU and donkey anti-rat Alexa 488 secondary antibody (Thermo Fisher Scientific Cat# A-21208, RRID:AB_2535794). DNA fibers were visualized using a Zeiss LSM 5 Pascal confocal microscope and processed with ImageJ software (ImageJ 1.52a).

### Protein analysis

For western blot analysis, samples were prepared by direct lysis in Laemmli buffer. Antibodies used were: anti-actin (Sigma-Aldrich Cat# A2066, RRID:AB_476693), anti-Ku70 (Santa Cruz Biotechnology Cat# sc-5309, RRID:AB_628453), anti-PCNA (Santa Cruz Biotechnology Cat# sc-56, RRID:AB_628110), anti-Ubiquityl-PCNA (Cell Signaling Technology Cat# 13439, RRID:AB_2798219), anti-p21 (C-19, Santa Cruz Biotechnology Cat# sc-397, RRID: AB_632126), anti-myc (Bethyl Cat# A190-105A, RRID: AB_67390). Secondary antibodies were from Sigma, and detection was performed with ECL according to the manufacturer’s instructions (Amersham GE Healthcare). When using secondary fluorescence antibodies: Invitrogen, anti-rabbit (Thermo Fisher Scientific Cat# A32808, RRID: AB_2762837), anti-mouse (Thermo Fisher Scientific Cat# A32789, RRID: AB_2762832). Western blot images were acquired with Image Quant^TM^ LAS4000 (GE, Healthcare ImageQuant, LAS 4000 v 1.0) and Odyssey CLx Imager and processed with ImageJ software (ImageJ 1.52a). For protein half-life assays, U2OS cells were cultured at 80% confluence in 12-well culture plates with 25 µg/mL cycloheximide (CHX), and incubated for different time points ranging from 30 min to 8 h. They were then lysed and analyzed as described before. Raw western blot data can be found in Figs. S8 and S9.

### Cell survival/viability assays

Cells were plated in 12 or 6 well-plates to obtain 70% confluence 24 h later. The next day, cells were transfected and/or transduced and 24 h after transfection/transduction, cells were harvested and re-plated at low confluence in a 96-well plate in order to get a 90% confluence at the end point for the assay. Each condition had three technical replicates. Twenty-four hours later, cells remained untreated or were treated with CDDP, UV, Chk1i, ATRi, Wee1i, HU or Ola. Treatment times and concentrations for each experiment, are specified in the figure legends. After treatments, cells were fixed with 4% PFA/2% sucrose for 20 min. DAPI staining served to visualize nuclei. INCell 2200 and INCell Analyzer WorkStations were used to obtain images and count nuclei, respectively. In some cell lines, after treatments, cell viability was measured with the use of CellTiter-Glo® Assay (Promega Cat # G7571) according to the manufacturer’s protocol. Luminescence was measured with Varioskan™ LUX (Thermo Scientific).

### SYTOX Green staining

U2OS cells were plated on 35 mm culture plates and treated according to the experimental scheme. Subsequently, the cells were trypsinized, recovered from the culture plates in 15 mL falcon tubes and centrifuged at 1500 rpm for 5 min. The supernatant was removed, the pellet was resuspended in PBS and the samples were transferred to cytometry tubes. The necessary amount of SYTOX^TM^ Green was then added to reach a final concentration of 10 nM and the tubes were incubated for at least 20 min on ice and protected from light. Samples were analyzed on a FACS Aria Fusion cytometer using the 488 laser with a 530/30 filter.

### Statistical analysis

Statistical analysis was performed using two software tools: GraphPad Prism 8 and Infostat. Frequency distributions of DNA track lengths and ratios were calculated using GraphPad Prism 8 software. All statistical analyses were performed using appropriate tests based on the distribution and variance characteristics of the data. Prior to conducting the main analyses, the assumption of normality and homogeneity of variances were assessed. Depending on the results of these preliminary tests and the type of tests used for these experiments in bibliography, parametric or non-parametric tests were selected. When the data generated non-Gaussian distributions, the statistical analyses used to compare two and more than two variables were the Mann–Whitney and Kruskal–Wallis tests, respectively. In all the analyses shown in this study, each letter groups samples that are similar (no significant difference) while samples with different letters are significantly different. Hence, two samples identified with the same letter are not significantly different between each other. In contrast, samples that do not share any letter are significantly different. *p* < 0.001 was considered significant for frequency distribution in fiber analysis; PLA; myc, p21 and BrdU intensity. *p* < 0.05 was considered significant for plots showing the mean of independent experiments. Sample size was defined as the smallest number of events that provides a result that does not vary when increasing the sample size. In addition, we verified that the sample size in this study is in the same range as the one reported in other published manuscripts evaluating the same variables. Information on sample sizes is provided for each experiment in the figure legends and supplementary figure legends. No exclusion criteria were used (only entire experiments that had technical issues were discarded but within analyzed samples, no events were excluded).

## Supplementary information

Supplemental Material

Supplemental Material

## Data Availability

All data generated or analyzed during this study are included in this published article (and its Supplementary Information files). All Western blot membranes used to assemble figures in this article are shown in Supplementary Figs. 8 and 9. Source data from all datasets analyzed during the current study are listed in an Excel file in the Supplementary information section.

## References

[1] Bertolin AP, Mansilla SF, Gottifredi V. The identification of translesion DNA synthesis regulators: inhibitors in the spotlight. DNA Repair. 2015;32:158–64. 26002196 10.1016/j.dnarep.2015.04.027 PMC4522396

[2] Tonzi P, Huang TT. Role of Y-family translesion DNA polymerases in replication stress: implications for new cancer therapeutic targets. DNA Repair. 2019;78:20–6. 30954011 10.1016/j.dnarep.2019.03.016 PMC6534436

[3] Guo C, Sonoda E, Tang TS, Parker JL, Bielen AB, Takeda S, et al. REV1 protein interacts with PCNA: significance of the REV1 BRCT domain in vitro and in vivo. Mol Cell. 2006;23:265–71. 16857592 10.1016/j.molcel.2006.05.038

[4] Bienko M, Green CM, Crosetto N, Rudolf F, Zapart G, Coull B, et al. Ubiquitin-binding domains in Y-family polymerases regulate translesion synthesis. Science. 2005;310:1821–4. 16357261 10.1126/science.1120615

[5] Edmunds CE, Simpson LJ, Sale JE. PCNA ubiquitination and REV1 define temporally distinct mechanisms for controlling translesion synthesis in the avian cell line DT40. Mol Cell. 2008;30:519–29. 18498753 10.1016/j.molcel.2008.03.024

[6] Kannouche PL, Wing J, Lehmann AR. Interaction of human DNA polymerase eta with monoubiquitinated PCNA: a possible mechanism for the polymerase switch in response to DNA damage. Mol Cell. 2004;14:491–500. 15149598 10.1016/s1097-2765(04)00259-x

[7] Tissier A, Kannouche P, Reck MP, Lehmann AR, Fuchs RP, Cordonnier A. Co-localization in replication foci and interaction of human Y-family members, DNA polymerase pol eta and REVl protein. DNA Repair. 2004;3:1503–14. 15380106 10.1016/j.dnarep.2004.06.015

[8] Ogi T, Kannouche P, Lehmann AR. Localisation of human Y-family DNA polymerase kappa: relationship to PCNA foci. J Cell Sci. 2005;118:129–36. 15601657 10.1242/jcs.01603

[9] Lerner LK, Francisco G, Soltys DT, Rocha CR, Quinet A, Vessoni AT, et al. Predominant role of DNA polymerase eta and p53-dependent translesion synthesis in the survival of ultraviolet-irradiated human cells. Nucleic Acids Res. 2017;45:1270–80. 28180309 10.1093/nar/gkw1196 PMC5388406

[10] Bi X, Slater DM, Ohmori H, Vaziri CDNA. polymerase kappa is specifically required for recovery from the benzo[a]pyrene-dihydrodiol epoxide (BPDE)-induced S-phase checkpoint. J Biol Chem. 2005;280:22343–55. 15817457 10.1074/jbc.M501562200

[11] Hendel A, Ziv O, Gueranger Q, Geacintov N, Livneh Z. Reduced efficiency and increased mutagenicity of translesion DNA synthesis across a TT cyclobutane pyrimidine dimer, but not a TT 6-4 photoproduct, in human cells lacking DNA polymerase eta. DNA Repair. 2008;7:1636–46. 18634905 10.1016/j.dnarep.2008.06.008 PMC2656611

[12] Klarer AC, Stallons LJ, Burke TJ, Skaggs RL, McGregor WG. DNA polymerase eta participates in the mutagenic bypass of adducts induced by benzo[a]pyrene diol epoxide in mammalian cells. PLoS ONE. 2012;7:e39596. 22745795 10.1371/journal.pone.0039596 PMC3380003

[13] Federico MB, Siri SO, Calzetta NL, Paviolo NS, de la Vega MB, Martino J, et al. Unscheduled MRE11 activity triggers cell death but not chromosome instability in polymerase eta-depleted cells subjected to UV irradiation. Oncogene. 2020;39:3952–64. 32203168 10.1038/s41388-020-1265-9

[14] Srivastava AK, Han C, Zhao R, Cui T, Dai Y, Mao C, et al. Enhanced expression of DNA polymerase eta contributes to cisplatin resistance of ovarian cancer stem cells. Proc Natl Acad Sci USA. 2015;112:4411–6. 25831546 10.1073/pnas.1421365112 PMC4394248

[15] Maiorano D, El Etri J, Franchet C, Hoffmann JS. Translesion synthesis or repair by specialized DNA polymerases limits excessive genomic instability upon replication stress. Int J Mol Sci.2021;22:3924 33920223 10.3390/ijms22083924 PMC8069355

[16] Zafar MK, Eoff RL. Translesion DNA synthesis in cancer: molecular mechanisms and therapeutic opportunities. Chem Res Toxicol. 2017;30:1942–55. 28841374 10.1021/acs.chemrestox.7b00157 PMC7135728

[17] Patel SM, Dash RC, Hadden MK. Translesion synthesis inhibitors as a new class of cancer chemotherapeutics. Expert Opin Investig Drugs. 2021;30:13–24. 33179552 10.1080/13543784.2021.1850692 PMC7832080

[18] Chatterjee N, Whitman MA, Harris CA, Min SM, Jonas O, Lien EC, et al. REV1 inhibitor JH-RE-06 enhances tumor cell response to chemotherapy by triggering senescence hallmarks. Proc Natl Acad Sci USA. 2020;117:28918–21. 33168727 10.1073/pnas.2016064117 PMC7682577

[19] Chen Y, Jie X, Xing B, Wu Z, Yang X, Rao X, et al. REV1 promotes lung tumorigenesis by activating the Rad18/SERTAD2 axis. Cell Death Dis. 2022;13:110. 35115490 10.1038/s41419-022-04567-5 PMC8814179

[20] Mansilla SF, de la Vega MB, Calzetta NL, Siri SO, Gottifredi V. CDK-independent and PCNA-dependent functions of p21 in DNA replication. Genes. 2020;11:593 32481484 10.3390/genes11060593 PMC7349641

[21] Soria G, Speroni J, Podhajcer OL, Prives C, Gottifredi V. p21 differentially regulates DNA replication and DNA-repair-associated processes after UV irradiation. J Cell Sci. 2008;121:3271–82. 18782865 10.1242/jcs.027730

[22] Mansilla SF, Soria G, Vallerga MB, Habif M, Martinez-Lopez W, Prives C, et al. UV-triggered p21 degradation facilitates damaged-DNA replication and preserves genomic stability. Nucleic Acids Res. 2013;41:6942–51. 23723248 10.1093/nar/gkt475 PMC3737556

[23] Soria G, Podhajcer O, Prives C, Gottifredi V. P21Cip1/WAF1 downregulation is required for efficient PCNA ubiquitination after UV irradiation. Oncogene. 2006;25:2829–38. 16407842 10.1038/sj.onc.1209315

[24] Bloom J, Amador V, Bartolini F, DeMartino G, Pagano M. Proteasome-mediated degradation of p21 via N-terminal ubiquitinylation. Cell. 2003;115:71–82. 14532004 10.1016/s0092-8674(03)00755-4

[25] Havens CG, Walter JC. Docking of a specialized PIP Box onto chromatin-bound PCNA creates a degron for the ubiquitin ligase CRL4Cdt2. Mol Cell. 2009;35:93–104. 19595719 10.1016/j.molcel.2009.05.012 PMC2744448

[26] Horsfall AJ, Vandborg BA, Kowalczyk W, Chav T, Scanlon DB, Abell AD, et al. Unlocking the PIP-box: a peptide library reveals interactions that drive high-affinity binding to human PCNA. J Biol Chem. 2021;296:100773. 33984330 10.1016/j.jbc.2021.100773 PMC8191301

[27] Zheleva DI, Zhelev NZ, Fischer PM, Duff SV, Warbrick E, Blake DG, et al. A quantitative study of the in vitro binding of the C-terminal domain of p21 to PCNA: affinity, stoichiometry, and thermodynamics. Biochemistry. 2000;39:7388–97. 10858286 10.1021/bi992498r

[28] Cruet-Hennequart S, Gallagher K, Sokol AM, Villalan S, Prendergast AM, Carty MP. DNA polymerase eta, a key protein in translesion synthesis in human cells. Subcell Biochem. 2010;50:189–209. 20012583 10.1007/978-90-481-3471-7_10

[29] Jahjah T, Singh JK, Gottifredi V, Quinet A. Tolerating DNA damage by repriming: gap filling in the spotlight. DNA Repair. 2024;142:103758. 39236419 10.1016/j.dnarep.2024.103758

[30] Maya-Mendoza A, Moudry P, Merchut-Maya JM, Lee M, Strauss R, Bartek J. High speed of fork progression induces DNA replication stress and genomic instability. Nature. 2018;559:279–84. 29950726 10.1038/s41586-018-0261-5

[31] de Gruijl FR. Skin cancer and solar UV radiation. Eur J Cancer. 1999;35:2003–9. 10711242 10.1016/s0959-8049(99)00283-x

[32] Rastogi RP, Richa, Kumar A, Tyagi MB, Sinha RP. Molecular mechanisms of ultraviolet radiation-induced DNA damage and repair. J Nucleic Acids. 2010;2010:592980. 21209706 10.4061/2010/592980 PMC3010660

[33] Wang D, Lippard SJ. Cellular processing of platinum anticancer drugs. Nat Rev Drug Discov. 2005;4:307–20. 15789122 10.1038/nrd1691

[34] Koc A, Wheeler LJ, Mathews CK, Merrill GF. Hydroxyurea arrests DNA replication by a mechanism that preserves basal dNTP pools. J Biol Chem. 2004;279:223–30. 14573610 10.1074/jbc.M303952200

[35] Segeren HA, Westendorp B. Mechanisms used by cancer cells to tolerate drug-induced replication stress. Cancer Lett. 2022;544:215804. 35750276 10.1016/j.canlet.2022.215804

[36] Karnitz LM, Zou L. Molecular pathways: targeting ATR in cancer therapy. Clin Cancer Res. 2015;21:4780–5. 26362996 10.1158/1078-0432.CCR-15-0479 PMC4631635

[37] Qiu Z, Oleinick NL, Zhang J. ATR/CHK1 inhibitors and cancer therapy. Radiother Oncol. 2018;126:450–64. 29054375 10.1016/j.radonc.2017.09.043 PMC5856582

[38] Do K, Doroshow JH, Kummar S. Wee1 kinase as a target for cancer therapy. Cell Cycle. 2013;12:3159–64. 24013427 10.4161/cc.26062 PMC3865011

[39] Esposito F, Giuffrida R, Raciti G, Puglisi C, Forte S. Wee1 kinase: a potential target to overcome tumor resistance to therapy. Int J Mol Sci. 2021;22:10689 34639030 10.3390/ijms221910689 PMC8508993

[40] Ngoi NYL, Pham MM, Tan DSP, Yap TA. Targeting the replication stress response through synthetic lethal strategies in cancer medicine. Trends Cancer. 2021;7:930–57. 34215565 10.1016/j.trecan.2021.06.002 PMC8458263

[41] Nayak S, Calvo JA, Cong K, Peng M, Berthiaume E, Jackson J, et al. Inhibition of the translesion synthesis polymerase REV1 exploits replication gaps as a cancer vulnerability. Sci Adv. 2020;6:eaaz7808. 32577513 10.1126/sciadv.aaz7808 PMC7286678

[42] Gagou ME, Zuazua-Villar P, Meuth M. Enhanced H2AX phosphorylation, DNA replication fork arrest, and cell death in the absence of Chk1. Mol Biol Cell. 2010;21:739–52. 20053681 10.1091/mbc.E09-07-0618 PMC2828961

[43] Calzetta NL, González Besteiro MA, Gottifredi V. Mus81-Eme1-dependent aberrant processing of DNA replication intermediates in mitosis impairs genome integrity. Sci Adv. 2020;6:eabc8257 33298441 10.1126/sciadv.abc8257 PMC7725468

[44] Syljuasen RG, Sorensen CS, Hansen LT, Fugger K, Lundin C, Johansson F, et al. Inhibition of human Chk1 causes increased initiation of DNA replication, phosphorylation of ATR targets, and DNA breakage. Mol Cell Biol. 2005;25:3553–62. 15831461 10.1128/MCB.25.9.3553-3562.2005 PMC1084285

[45] González Besteiro MA, Gottifredi V. The fork and the kinase: a DNA replication tale from a CHK1 perspective. Mutat Res. Rev. 2015;763:168–80. 10.1016/j.mrrev.2014.10.003 PMC4369321 25795119

[46] Maya-Mendoza A, Petermann E, Gillespie DA, Caldecott KW, Jackson DA. Chk1 regulates the density of active replication origins during the vertebrate S phase. EMBO J. 2007;26:2719–31. 17491592 10.1038/sj.emboj.7601714 PMC1888675

[47] Gonzalez Besteiro MA, Calzetta NL, Loureiro SM, Habif M, Betous R, Pillaire MJ, et al. Chk1 loss creates replication barriers that compromise cell survival independently of excess origin firing. EMBO J. 2019;38:e101284. 31294866 10.15252/embj.2018101284 PMC6694221

[48] Tsanov N, Kermi C, Coulombe P, Van der Laan S, Hodroj D, Maiorano D. PIP degron proteins, substrates of CRL4Cdt2, and not PIP boxes, interfere with DNA polymerase eta and kappa focus formation on UV damage. Nucleic Acids Res. 2014;42:3692–706. 24423875 10.1093/nar/gkt1400 PMC3973308

[49] Hishiki A, Hashimoto H, Hanafusa T, Kamei K, Ohashi E, Shimizu T, et al. Structural basis for novel interactions between human translesion synthesis polymerases and proliferating cell nuclear antigen. J Biol Chem. 2009;284:10552–60. 19208623 10.1074/jbc.M809745200 PMC2667742

[50] Gu L, Li M, Li CM, Haratipour P, Lingeman R, Jossart J, et al. Small molecule targeting of transcription-replication conflict for selective chemotherapy. Cell Chem Biol. 2023;30:1235–47.e6. 37531956 10.1016/j.chembiol.2023.07.001 PMC10592352

[51] Lemech CR, Kichenadasse G, Marschner JP, Alevizopoulos K, Otterlei M, Millward M. ATX-101, a cell-penetrating protein targeting PCNA, can be safely administered as intravenous infusion in patients and shows clinical activity in a Phase 1 study. Oncogene. 2023;42:541–4. 36564469 10.1038/s41388-022-02582-6 PMC9918429

[52] Muller R, Misund K, Holien T, Bachke S, Gilljam KM, Vatsveen TK, et al. Targeting proliferating cell nuclear antigen and its protein interactions induces apoptosis in multiple myeloma cells. PLoS ONE. 2013;8:e70430. 23936203 10.1371/journal.pone.0070430 PMC3729839

[53] Anand J, Chiou L, Sciandra C, Zhang X, Hong J, Wu D, et al. Roles of trans-lesion synthesis (TLS) DNA polymerases in tumorigenesis and cancer therapy. NAR Cancer. 2023;5:zcad005. 36755961 10.1093/narcan/zcad005 PMC9900426

[54] Gonzalez-Magana A, Blanco FJ. Human PCNA structure, function and interactions. Biomolecules. 2020;10:570. 32276417 10.3390/biom10040570 PMC7225939

[55] Wojtaszek JL, Chatterjee N, Najeeb J, Ramos A, Lee M, Bian K, et al. A small molecule targeting mutagenic translesion synthesis improves chemotherapy. Cell. 2019;178:152–9.e11. 31178121 10.1016/j.cell.2019.05.028 PMC6644000

[56] Yoon JH, Johnson RE, Prakash L, Prakash S. Implications of inhibition of Rev1 interaction with Y family DNA polymerases for cisplatin chemotherapy. Genes Dev. 2021;35:1256–70. 34385260 10.1101/gad.348662.121 PMC8415319

[57] Punchihewa C, Inoue A, Hishiki A, Fujikawa Y, Connelly M, Evison B, et al. Identification of small molecule proliferating cell nuclear antigen (PCNA) inhibitor that disrupts interactions with PIP-box proteins and inhibits DNA replication. J Biol Chem. 2012;287:14289–300. 22383522 10.1074/jbc.M112.353201 PMC3340206

[58] Gonzalez-Foutel NS, Glavina J, Borcherds WM, Safranchik M, Barrera-Vilarmau S, Sagar A, et al. Conformational buffering underlies functional selection in intrinsically disordered protein regions. Nat Struct Mol Biol. 2022;29:781–90. 35948766 10.1038/s41594-022-00811-w PMC10262780

[59] Rossino G, Marchese E, Galli G, Verde F, Finizio M, Serra M, et al. Peptides as therapeutic agents: challenges and opportunities in the green transition era. Molecules. 2023;28:7165. 37894644 10.3390/molecules28207165 PMC10609221

[60] Kannouche P, Broughton BC, Volker M, Hanaoka F, Mullenders LH, Lehmann AR. Domain structure, localization, and function of DNA polymerase eta, defective in xeroderma pigmentosum variant cells. Genes Dev. 2001;15:158–72. 11157773 10.1101/gad.187501 PMC312610

[61] Leonhardt H, Rahn HP, Weinzierl P, Sporbert A, Cremer T, Zink D, et al. Dynamics of DNA replication factories in living cells. J Cell Biol. 2000;149:271–80. 10769021 10.1083/jcb.149.2.271 PMC2175147

[62] Speroni J, Federico MB, Mansilla SF, Soria G, Gottifredi V. Kinase-independent function of checkpoint kinase 1 (Chk1) in the replication of damaged DNA. Proc Natl Acad Sci USA. 2012;109:7344–9. 22529391 10.1073/pnas.1116345109 PMC3358916

[63] Bienko M, Green CM, Sabbioneda S, Crosetto N, Matic I, Hibbert RG, et al. Regulation of translesion synthesis DNA polymerase eta by monoubiquitination. Mol Cell. 2010;37:396–407. 20159558 10.1016/j.molcel.2009.12.039

[64] Mansilla SF, Bertolin AP, Bergoglio V, Pillaire MJ, González Besteiro MA, Luzzani C, et al. Cyclin kinase-independent role of p21CDKN1A in the promotion of nascent DNA elongation in unstressed cells. eLife. 2016;5:e18020. 27740454 10.7554/eLife.18020 PMC5120883

[65] Harrigan JA, Belotserkovskaya R, Coates J, Dimitrova DS, Polo SE, Bradshaw CR, et al. Replication stress induces 53BP1-containing OPT domains in G1 cells. J Cell Biol. 2011;193:97–108. 21444690 10.1083/jcb.201011083 PMC3082192

