# Supplementary material for: Versatile enhancement of the killing potential of anti-cancer agents achieved by peptide mimetics of the PCNA interface towards specialized DNA polymerases
Source: Cell Death Dis. 2025 Jul 8;16(1):503. doi: 10.1038/s41419-025-07812-9 (PMC12238476; doi:10.1038/s41419-025-07812-9)
Supplement: Supplementary file 1 — Supplemental Material [file 41419_2025_7812_MOESM1_ESM.pdf]

Supplemental Material for

Versatile enhancement of the killing potential of anti-cancer agents achieved by peptide mimetics of the PCNA interface towards specialized DNA polymerases

Yiovana Verónica Okraine°[1], María Belén de la Vega°[1], Sofía Venerus Arbilla[1], Ginette Moyano[1], Agostina P. Bertolin[1,2], Horacio M. Pallarés[1,3], Lisa Wiesmüller[4], Sabrina F. Mansilla[1]*, Vanesa Gottifredi[1]*

1. Fundación Instituto Leloir, Consejo Nacional de Investigaciones Científicas y Técnicas (IIBBA), Buenos Aires 1405, CABA, Argentina
2. The Francis Crick Institute, NW1 1AT, London, UK
3. Stowers Institute, MO 64110, Kansas City, USA
4. Department of Obstetrics and Gynecology, Ulm University, Ulm 89075, Ulm, Germany

°equal contribution

* corresponding authors (smansilla@leloir.org.ar; vgottifredi@leloir.org.ar)

This PDF file includes:

Figs. S1 to S9 with accompanying figure legends. Original western blots were uploaded as Figures S8 and S9.

Legend for Source data excel file.

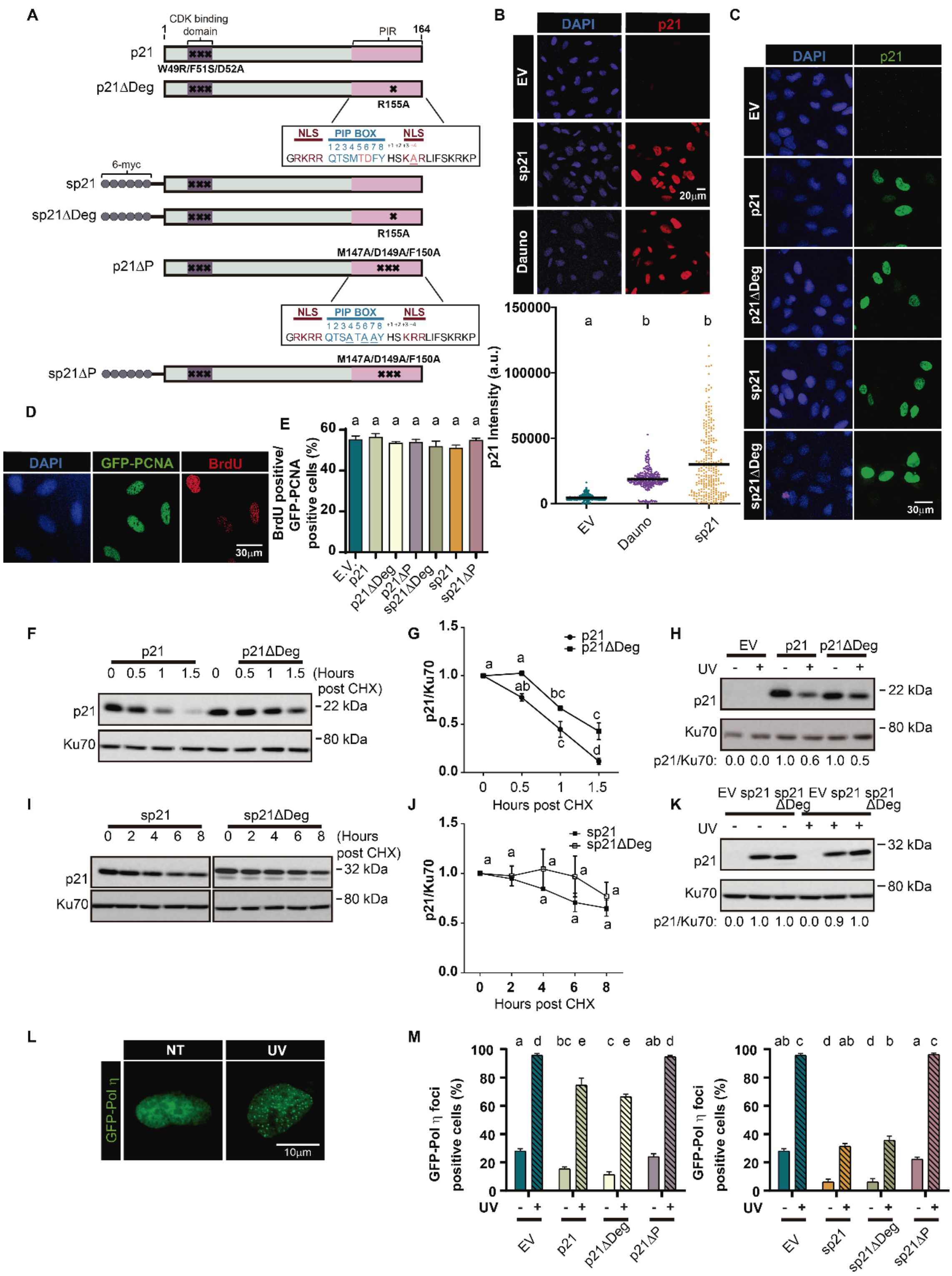

Figure S1: The degron sequence coupled to the PIR of p21 contributes to the stability of p21 in untreated conditions but it is not relevant for the inhibition of Pol η focal organization after UV.

A) Schematic representation of the p21 mutants used in this study. Three point mutations in the p21 sequence generate amino acid residue changes (W49R, F51S, D52A) that disrupt the interaction of p21 with cyclins/CDKs (23). The pink

box delimits the PCNA interacting region (PIR) of p21, which includes the nuclear localization signal (NLS) and the PCNA interacting motif called PIP box (blue letters). The PIP box is coupled with a degron sequence (pink letters) which contains a central threonine and aspartate (TD), relevant for the interaction with PCNA, and an arginine +4 (R+4) which is the docking site for the ubiquitin ligase CRL4-Cdt2 (25). The p21ΔDeg mutant was generated by introducing an R155A mutation. The N terminal 6-myc-tag depicted in the middle and lower panel, prevents p21 degradation after genotoxic stress such as the one caused by UV irradiation generating a stable version of the protein (sp21) (23). The interaction with PCNA is disrupted by the following changes in the amino acid sequence: M147A, D149A, and F150A (sp21ΔP) (23). For additional details about mutants used, see materials and methods section.

B) On top: Representative images of U2OS cells transfected with EV (empty vector) or sp21, or treated with Daunorubicin. Images show nuclei positive for p21 and DAPI staining. Notice that the levels of sp21 expression are comparable to those of endogenous p21 expression induced by Daunorubicin (0.22 μM for 24 h). Below: quantification of p21 intensity. A p21-specific antibody was used to detect p21. Median is shown in black. At least 260 nuclei were analyzed for each condition. N=2 (Statistics: Kruskal-Wallis, Dunn post-test).

C) Representative images of U2OS cells transfected with either EV, p21, p21ΔDeg, sp21 and sp21ΔDeg. Images show nuclei that stained positive when using p21 specific antibodies and DAPI staining.

D) Representative images of U2OS cells transfected with a GFP-tagged PCNA expression plasmid (GFP-PCNA) which was used to identify transfected cells. 24h after co-transfection of GFP-PCNA and the indicated plasmids, BrdU incorporation was performed for 15 min before fixation and denaturation. A BrdU-specific antibody and DAPI were used to detect replicating nuclei and DNA respectively.

E) Quantification of BrdU positive U2OS cells. The percentage (mean ± SD) of BrdU-positive cells was quantified after analyzing 700 nuclei positive for GFP-PCNA (which revealed the transfected population; percentage of colocalization: 80%) in three independent experiments (N=3) (Statistics: One-way ANOVA, Tukey post-test).

F) Western blot of U2OS cells transfected with the indicated plasmids and treated with cycloheximide (24 mg/ml), subjected to whole cell extraction at the indicated time points. Western blots were performed with anti-p21 specific

antibodies. Ku70 was used as loading control. A representative image is shown. N=2.

G) Quantification (mean ± SD) of the p21/Ku70 ratio from p21 and p21ΔDeg U2OS-transfected samples shown in F (Statistics: One-way ANOVA, Tukey post-test).

H) Western blot of U2OS cells transfected with the indicated plasmids, non-treated or irradiated with UV (40 J/m^2) as indicated and subjected to whole cell extraction 6 hours after the UV treatment. Western blots were performed with anti-p21 specific antibodies. Ku70 was used as a loading control. A representative image is shown. N=2.

I) Western blots of U2OS cells transfected with the indicated plasmids and treated with cycloheximide (24 mg/ml), subjected to whole cell extraction at the indicated time points. Western blots were performed using p21 specific antibodies. Ku70 was used as loading control. A representative image is shown. N=2.

J) Quantification (mean ± SD) of the p21/Ku70 ratio from sp21 and sp21ΔDeg samples shown in I (Statistics: One-way ANOVA, Tukey post-test).

K) Western blot of U2OS cells transfected with the indicated plasmids, non-treated or irradiated with UV (40 J/m^2) subjected to whole cell extraction 6 h after the UV treatment. Western blots of the indicated samples were performed with anti-p21 specific antibodies. Ku70 was used as a loading control. A representative image is shown. N=2.

L) Representative images of U2OS nuclei transfected with GFP-Pol η. Non-treated condition, without focal organization (NT) or UV-treated (UV 40 J/m^2) with focal organization are shown.

M) Quantification of the percentage (mean ± SD) of U2OS cells with nuclear foci organization of GFP-Pol η. Samples were co-transfected with GFP-Pol η and the indicated p21 mutants. Cells were fixed 24 h after transfection and they were either left untreated or exposed to UV irradiation (40 J/m^2) or fixed 4 h later. At least 200 GFP-positive cells/sample were analyzed. EV samples are the same for the two plots. N=3 (Statistics: One-way ANOVA, Tukey post-test).

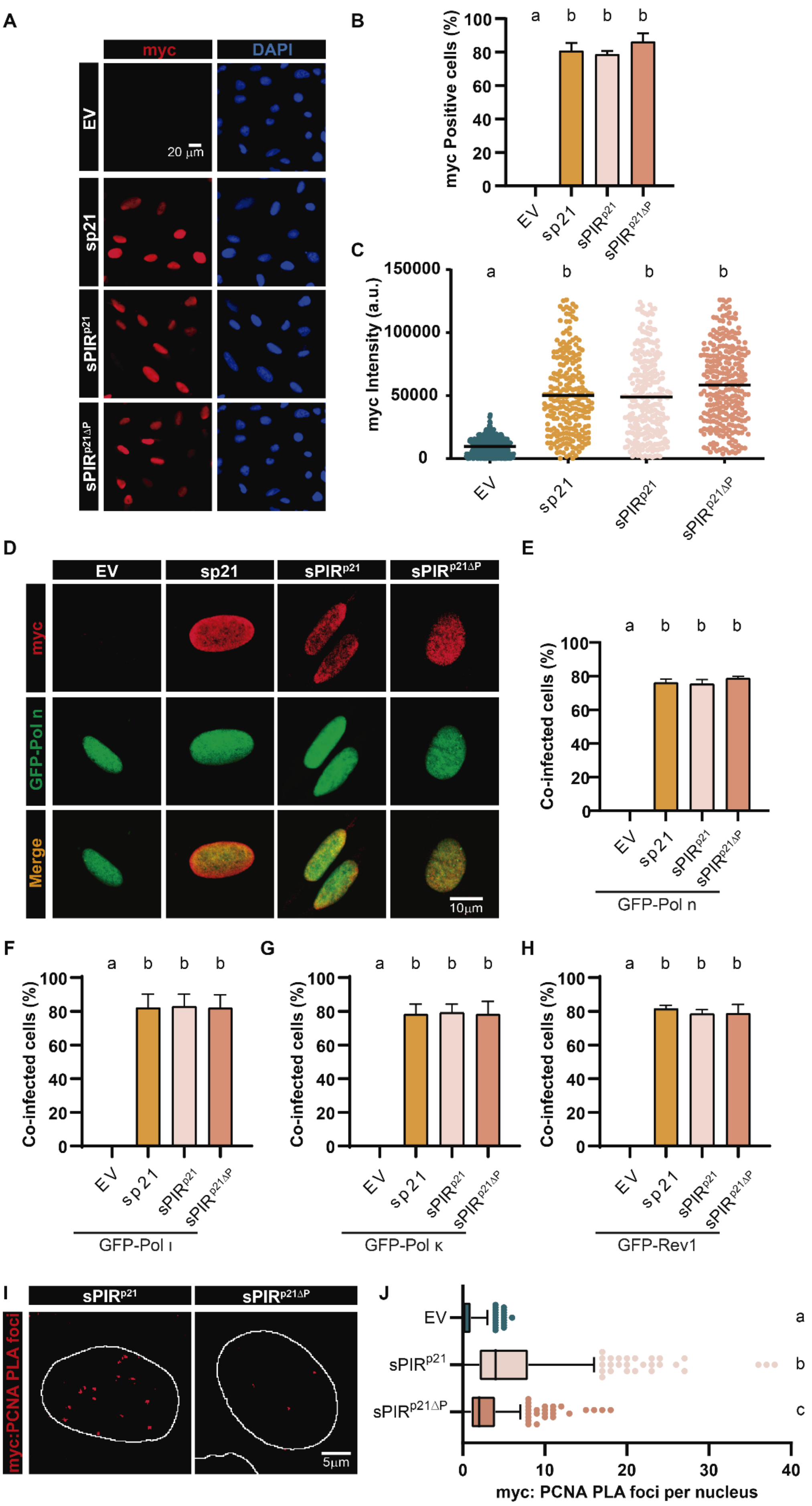

A
myc
DAPI
EV
sp21
sPIRp21
sPIRp21ΔP
20 µm
B
myc Positive cells (%)
100
80
60
40
20
0
a b b b
EV sp21 sPIRp21 sPIRp21ΔP
C
myc Intensity (a.u.)
150000
100000
50000
0
a b b b
EV sp21 sPIRp21 sPIRp21ΔP
D
EV sp21 sPIRp21 sPIRp21ΔP
myc
GFP-Pol η
Merge
10µm
E
Co-infected cells (%)
a b b b
EV sp21 sPIRp21 sPIRp21ΔP
GFP-Pol η
F
Co-infected cells (%)
a b b b
EV sp21 sPIRp21 sPIRp21ΔP
GFP-Pol ι
G
Co-infected cells (%)
a b b b
EV sp21 sPIRp21 sPIRp21ΔP
GFP-Pol κ
H
Co-infected cells (%)
a b b b
EV sp21 sPIRp21 sPIRp21ΔP
GFP-Rev1
I
sPIRp21
sPIRp21ΔP
myc:PCNA PLA foci
5µm
J
EV a
sPIRp21 b
sPIRp21ΔP c
0 10 20 30 40
myc: PCNA PLA foci per nucleus

Fig. S2: Transient delivery of p21 mutants by transduction is efficient and allows colocalization of p21 and transfected GFP-Pols.

- **A)** Representative images of U2OS cells transduced with the indicated vectors. Detection of p21 variants was performed by immunofluorescence using a specific myc-tag antibody.
- **B)** Percentage (mean ± SD) of cells expressing the indicated p21 mutants. U2OS cells were transduced and fixed 48 h later. At least 300 nuclei/sample were analyzed. N=3 (Statistics: One-way ANOVA, Tukey post-test).
- **C)** Quantification of myc intensity of the experiments shown in A and B. Median is shown in black. At least 225 nuclei were analyzed per condition. N=2 (Statistics: Kruskal-Wallis, Dunn post-test).
- **D)** Representative images of U2OS cells transfected with GFP-Pol η and transduced with the indicated vectors. Detection of the indicated p21 mutants was performed by immunofluorescence using a specific myc-tag antibody.
- **E)** Percentage (mean ± SD) of U2OS cells expressing both transfected GFP-Pol η and p21 transduced variants. Samples were transfected with GFP-Pol η and 5 h later transduced with the indicated viral particles. At least 500 nuclei/sample were analyzed. N=3 (Statistics: One-way ANOVA, Tukey post-test).
- **F)** Percentage (mean ± SD) of U2OS cells expressing both transfected GFP-Pol ι and p21 transduced variants. Samples were transfected with GFP-Pol η and 5 h later transduced with the indicated viral particles. At least 500 nuclei/sample were analyzed. N=2 (Statistics: One-way ANOVA, Tukey post-test).
- **G)** Percentage (mean ± SD) of U2OS cells expressing both transfected GFP-Pol κ and p21 transduced variants. Samples were transfected with GFP-Pol η and 5 h later transduced with the indicated viral particles. At least 500 nuclei/sample were analyzed. N=2 (Statistics: One-way ANOVA, Tukey post-test).
- **H)** Percentage (mean ± SD) of U2OS cells expressing both transfected GFP-Rev1 and p21 transduced variants. Samples were transfected with GFP-Pol η and 5 h later transduced with the indicated viral particles. At least 500 nuclei/sample were analyzed. N=2 (Statistics: One-way ANOVA, Tukey post-test).
- **I)** Representative images of U2OS cells transduced with the indicated vectors. Detection of myc:PCNA PLA foci was carried out using specific antibodies against myc and PCNA generated in different species, as well as PLA probe kits. White line delimits the nuclei area. DAPI channel data was used to create a mask in order to generate the white line.

J) PLA foci per nucleus (box and whiskers plot with 5-95% confidence interval) of U2OS cells shown in I, expressing transduced p21 variants. At least 500 nuclei/sample were analyzed. N=2 (Statistics: Kruskal-Wallis, Dunn post-test).

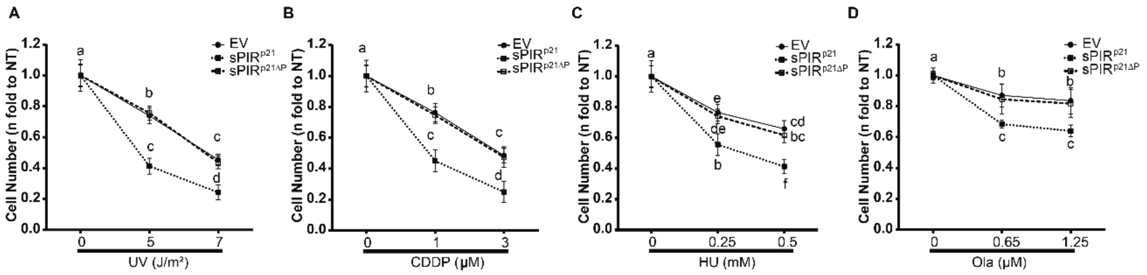

Fig. S3: The sPIR^{p21} synergizes with DNA damaging agents in preventing amplification of cancer cells.

- **A)** Cell number (mean ± SD) relative to untreated U2OS cells transduced with either EV, sPIR^{p21} or sPIR$^{p21\Delta P}$. 6 days after irradiation with the indicated UV dose, the total cell number in 3 wells from a 96-well plate was counted for each condition. Data shown for EV NT condition is the same for panels A to D. N=3 (Statistics: One-way ANOVA, Tukey post-test). The source data used was also used in Figure 4B.
- **B)** Cell number (mean ± SD) relative to untreated U2OS cells transduced with either EV, sPIR^{p21} or sPIR$^{p21\Delta P}$. 6 days after treatment with the indicated CDDP dose, the total cell number in 3 wells from a 96-well plate was counted for each condition. N=3 (Statistics: One-way ANOVA, Tukey post-test). The source data used was also used in Figure 4B.
- **C)** Cell number (mean ± SD) relative to untreated U2OS cells transduced with either EV, sPIR^{p21} or sPIR$^{p21\Delta P}$. 6 days after treatment with the indicated HU dose, the total cell number in 3 wells from a 96-well plate was counted for each condition. N=3 (Statistics: One-way ANOVA, Tukey post-test). The source data used was also used in Figure 4B.
- **D)** Cell number (mean ± SD) relative to untreated U2OS cells transduced with either EV, sPIR^{p21} or sPIR$^{p21\Delta P}$. 6 days after treatment with the indicated Ola dose, the total cell number in 3 wells from a 96-well plate was counted for each condition. N=3 (Statistics: One-way ANOVA, Tukey post-test). The source data used was also used in Figure 4B.

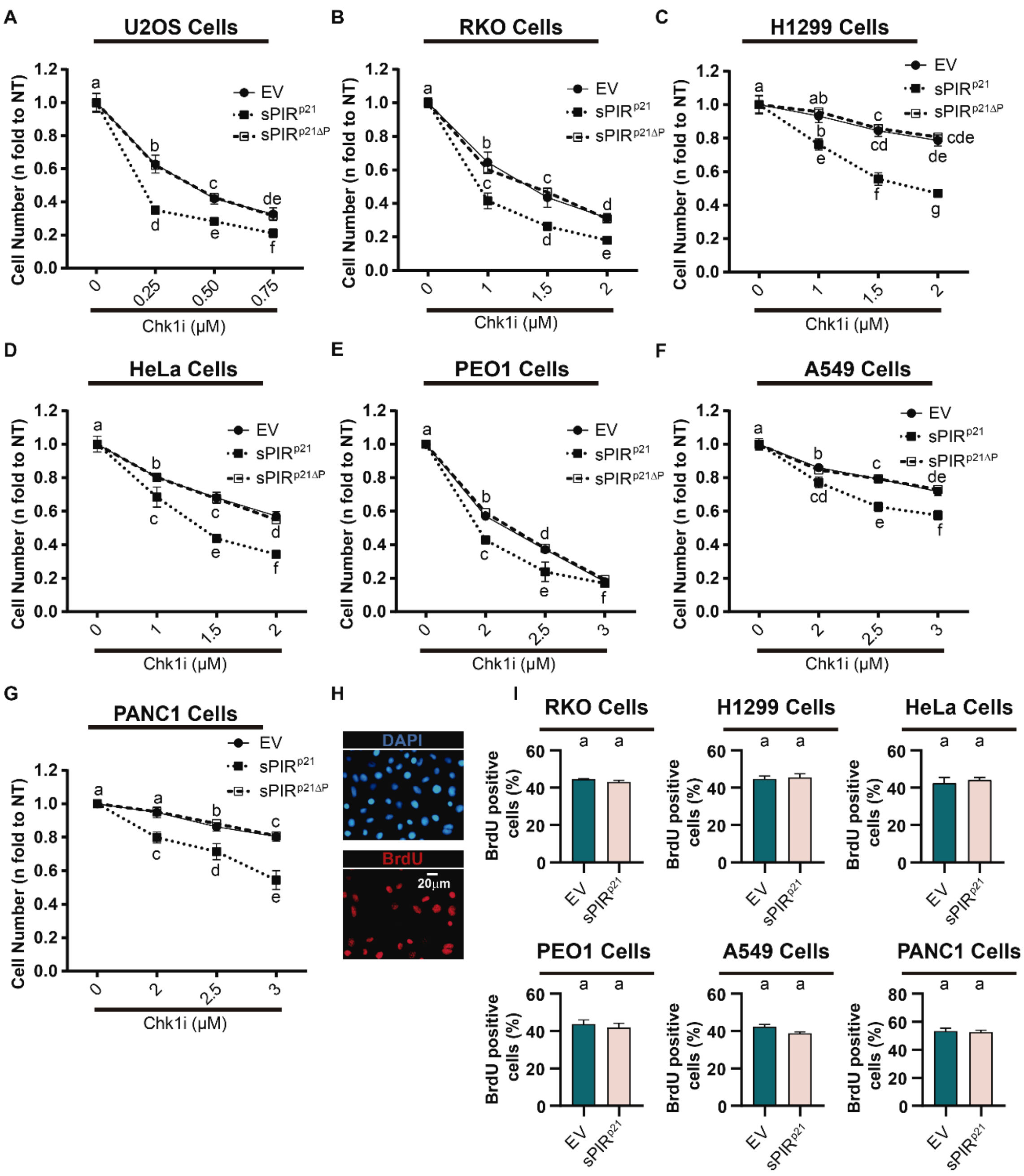

Fig. S4: The sPIR^{p21} sensitizes different cell lines to Chk1 inhibitors.

A) Cell number (mean ± SD) relative to untreated U2OS samples transduced with either EV, sPIR^{p21} or sPIR$^{p21\Delta P}$. 6 days after treatment with increasing doses of Chk1i, the total cell number in 3 wells from a 96-well plate was determined for each condition. N=3 (Statistics: One-way ANOVA, Tukey post-test).

B) Cell number (mean ± SD) relative to untreated RKO samples transduced with either EV, sPIR^{p21} or sPIR$^{p21\Delta P}$. 6 days after treatment with increasing doses of Chk1i, the total cell number in 3 wells from a 96-well plate was determined for each condition. N=3 (Statistics: One-way ANOVA, Tukey post-test). The source data was also used in Fig. 5D.

C) Cell number (mean ± SD) relative to untreated H1299 samples transduced with either EV, $sPIR^{p21}$ or $sPIR^{p21\Delta P}$. 6 days after treatment with increasing doses of Chk1i, the total cell number in 3 wells from a 96-well plate was determined for each condition. N=3 (Statistics: One-way ANOVA, Tukey post-test). The source data was also used in Fig. 5F.

D) Cell number (mean ± SD) relative to untreated Hela samples transduced with either EV, $sPIR^{p21}$ or $sPIR^{p21\Delta P}$. 6 days after treatment with increasing doses of Chk1i, the total cell number in 3 wells from a 96-well plate was determined for each condition. N=3 (Statistics: One-way ANOVA, Tukey post-test). The source data was also used in Fig. 5E.

E) Cell number (mean ± SD) relative to untreated PEO1 samples transduced with either EV, $sPIR^{p21}$ or $sPIR^{p21\Delta P}$. 6 days after treatment with increasing doses of Chk1i, the total cell number in 3 wells from a 96-well plate was determined for each condition. N=3 (Statistics: One-way ANOVA, Tukey post-test). The source data was also used in Fig. 5I.

F) Cell number (mean ± SD) relative to untreated A549 samples transduced with either EV, $sPIR^{p21}$ or $sPIR^{p21\Delta P}$. 6 days after treatment with increasing doses of Chk1i, the total cell number in 3 wells from a 96-well plate was determined for each condition. N=3 (Statistics: One-way ANOVA, Tukey post-test). The source data was also used in Fig. 5G.

G) Cell number (mean ± SD) relative to untreated PANC1 samples transduced with either EV, $sPIR^{p21}$ or $sPIR^{p21\Delta P}$. 6 days after treatment with increasing doses of Chk1i, the total cell number in 3 wells from a 96-well plate was determined for each condition. N=3 (Statistics: One-way ANOVA, Tukey post-test). The source data was also used in 5H.

H) Representative fields of U2OS cells pulse-labeled with BrdU for 15 min before fixation and denaturation.

I) Percentage (mean ± SD) of BrdU positive cells transduced with EV or $sPIR^{p21}$. At least 300 cells/sample were analyzed in total. N=3 (Statistics: Student t-test).

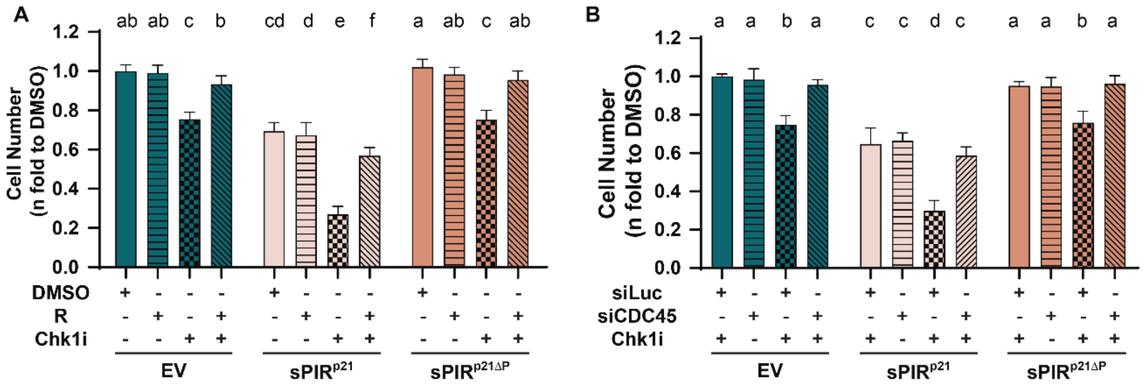

Fig. S5: sPIR^{p21} but not EV or sPIR$^{p21\Delta P}$ synergizes with Chk1i in killing cancer cells and roscovitine and CDC45 depletion revert such cytotoxic effect.

- **A)** Cell number (mean ± SD) of U2OS cells transduced with EV, sPIR^{p21}, sPIR$^{p21\Delta P}$ and fixed 6 days after treatment with DMSO as control or with Chk1i (0.5 µM), roscovitine (2.5 µM) or both (Chk1i + roscovitine). Each condition was relativized to EV-DMSO control. The total cell number in 3 wells from a 96-well plate was counted for each condition. N=3 (Statistics: One-way ANOVA, Tukey post-test). The source data was also used in Figure 6K.
- **B)** Cell number (mean ± SD) relative to EV-siLuc untreated U2OS cells. Samples were transfected with siLuc or siCDC45 and 5 hours later transduced with EV, sPIR^{p21} or sPIR$^{p21\Delta P}$, mock treated (NT) or treated with Chk1i (0.5 µM) for 6 days. The total cell number in 3 wells from a 96-well plate was counted for each condition. N=3 (Statistics: One-way ANOVA, Tukey post-test). The source data was also used in Figure 6L.

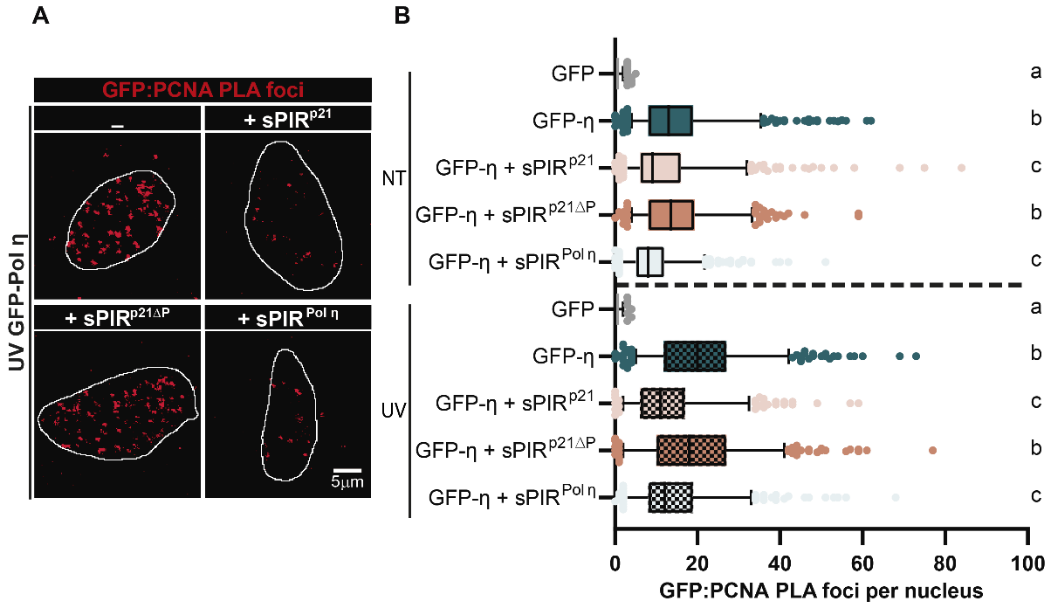

Fig. S6: sPIR^{p21} and sPIR$^{Pol\ \eta}$, but not mutants defective in PCNA binding, impair PCNA-Pol η interaction.

A) Representative images of U2OS cells transfected and transduced with the indicated vectors, UV-treated (40 J/m^2 for 4 h) and pre-extracted with ice-cold 0.5% Triton X-100 in CSK buffer before fixation. Detection of GFP:PCNA PLA foci was carried out using specific antibodies against GFP and PCNA generated in different species, as well as PLA probe kits. White line delimits the nuclei area. DAPI channel data was used to create a mask in order to generate the white line.

B) PLA foci per nucleus (box and whiskers plot with 5-95% confidence interval) of U2OS cells shown in A, expressing both transfected GFP or GFP-Pol η and transduced small peptide variants and either mock (NT) or UV-treated (40 J/m^2 for 4 h). Cells were pre-extracted with ice-cold 0.5% Triton X-100 in CSK buffer before fixation. At least 350 nuclei/sample were analyzed. N=2 (Statistics: Kruskal-Wallis, Dunn post-test, samples were compared within each treatment).

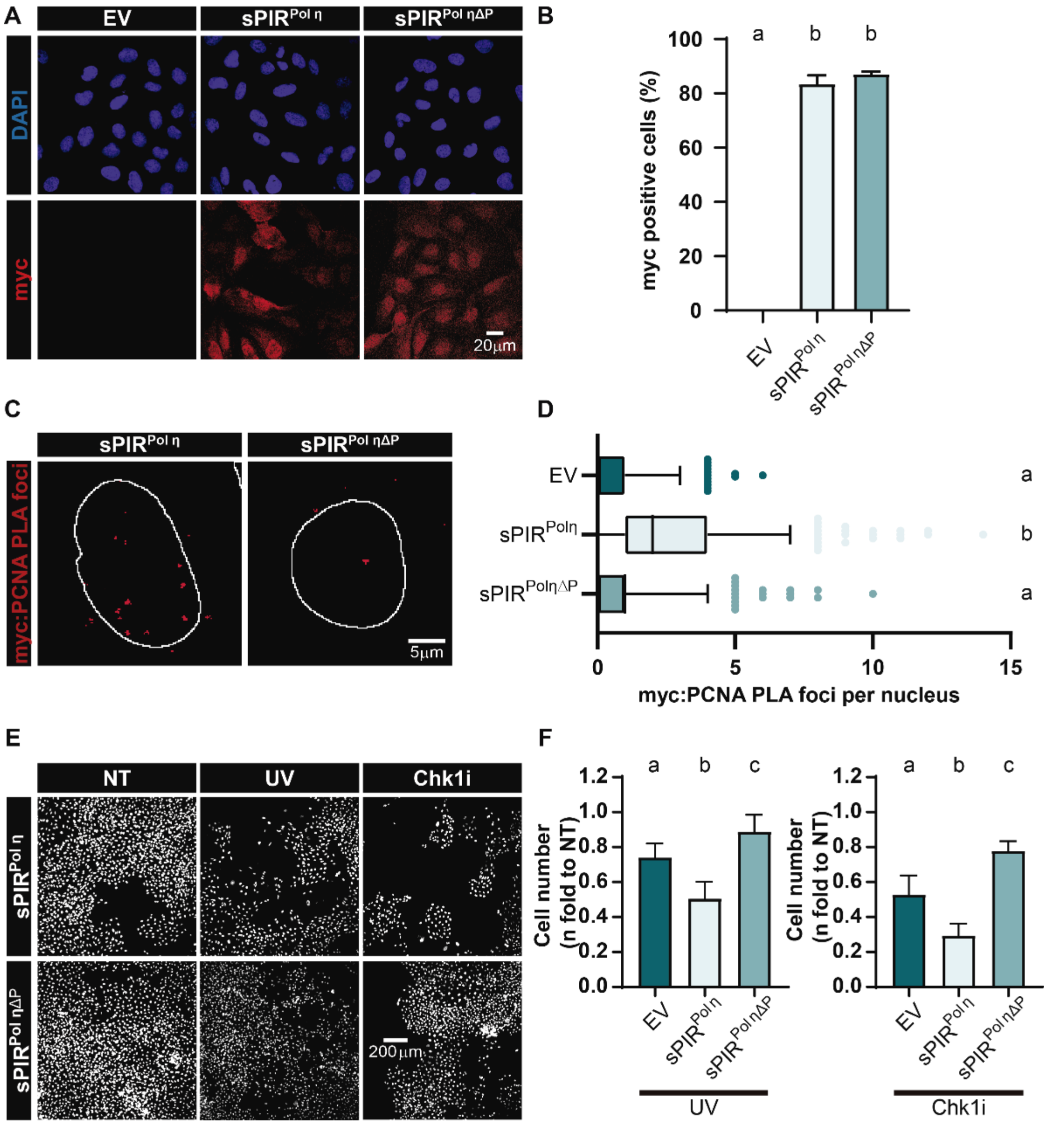

Fig. S7: sPIR$^{Pol\ \eta}$ but not sPIR$^{Pol\ \eta\Delta P}$ synergizes with UV and Chk1i in augmenting their cytotoxicity.

- **A)** Representative images of U2OS cells transduced with the indicated vectors. Detection of Pol η variants was performed by immunofluorescence using a specific myc-tag antibody.
- **B)** Percentage (mean ± SD) of cells expressing the indicated sPIR$^{Pol\ \eta}$ variants. U2OS cells were transduced and fixed 48 h later. At least 300 nuclei/sample were analyzed. N=2 (Statistics: One-way ANOVA, Tukey post-test).
- **C)** Representative images of U2OS cells transduced with the indicated vectors. Detection of myc:PCNA PLA foci was carried out using specific antibodies against myc and PCNA generated in different species, as well as PLA probe kits. White line delimits the nuclei area. DAPI channel data was used to create a mask in order to generate the white line.

D) PLA foci per nucleus (box and whiskers plot with 5-95% confidence interval) of U2OS cells shown in C, expressing transduced $sPIR^{Pol\ \eta}$ variants. At least 500 nuclei/sample were analyzed. N=2 (Statistics: Kruskal-Wallis, Dunn post-test).

E) Representative images of DAPI-stained U2OS cells transduced with either EV or $sPIR^{Pol\ \eta}$ particles. 48 h later, cells were either mock treated (NT) or treated with UV (7 J/m^2) or Chk1i (Gö6976, 0.5 µM) and fixed 6 days later.

F) Cell number (mean ± SD) expressed as fold changes with respect to the EV transduced, mock treated (NT) U2OS cells shown in E. The total cell number in 3 wells from a 96-well plate was quantified for each condition. N=2 (Statistics: One-way ANOVA, Tukey post-test).

Fig. 1B

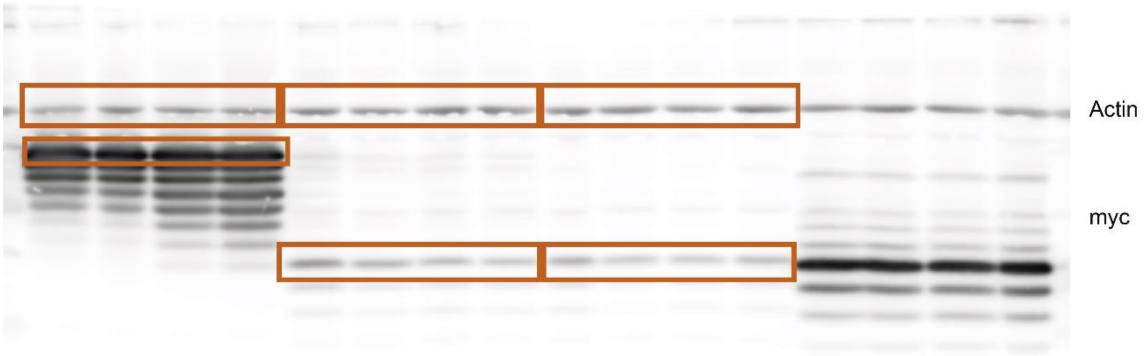

Fig. 1G

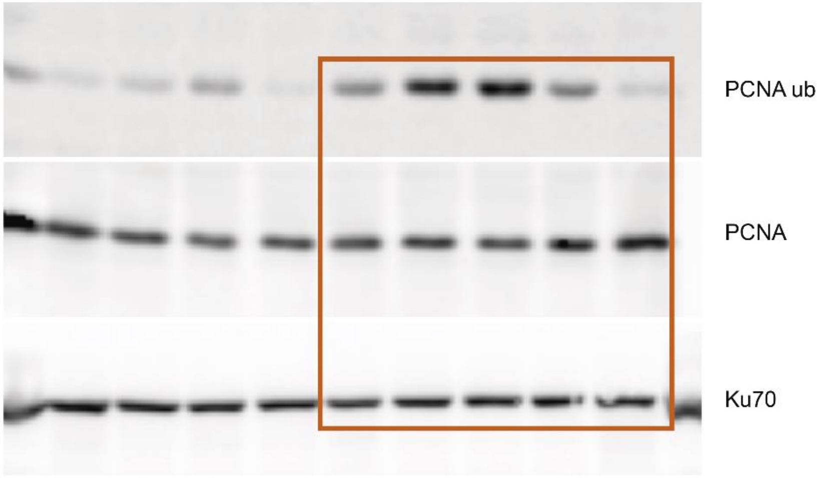

Fig. 3A

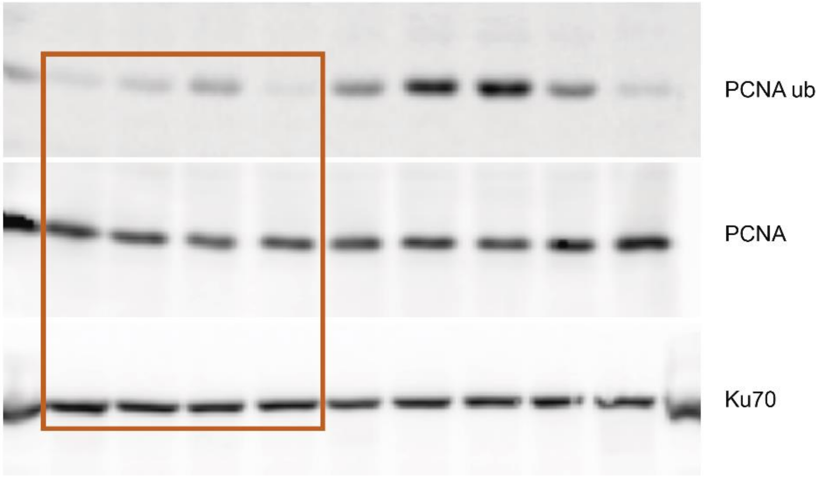

Fig. S8: Source data I.

Original uncropped images of Western Blot membranes used in the main figures of this manuscript.

Fig. S1F

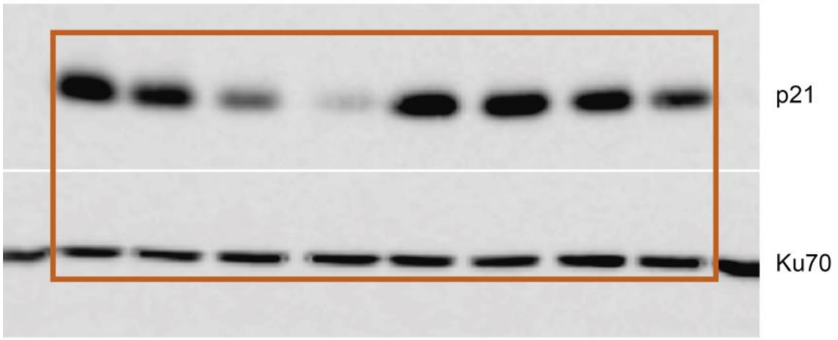

Fig. S1H

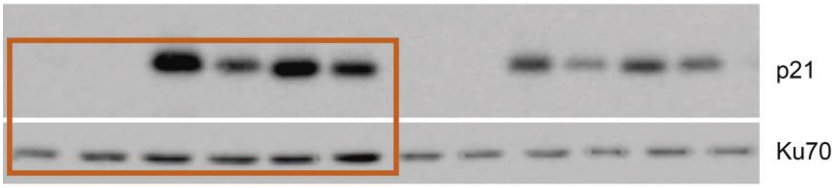

Fig. S1I

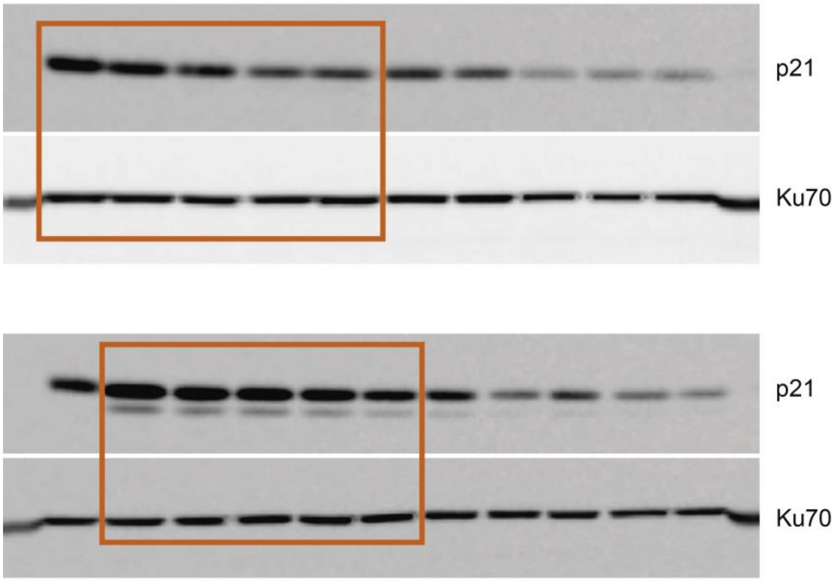

Fig. S1K

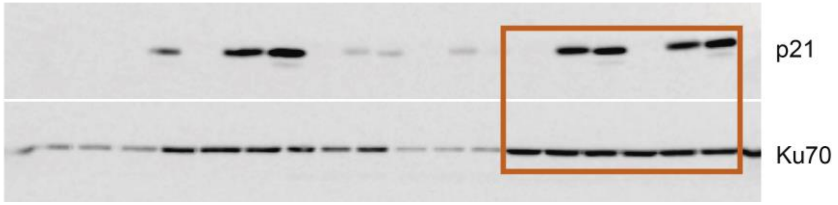

Fig. S9: Source data II.

Original uncropped images of Western Blot membranes used in the supplementary figures of this manuscript.

Source data excel file: Excel sheets containing the raw data used in all figures of this manuscript. A list of the authors responsible for each panel is included in the first tab.
